# Visuo-spatial imagery in dreams of congenitally and early blind: a systematic review

**DOI:** 10.3389/fnint.2023.1204129

**Published:** 2023-06-30

**Authors:** Katarina Ilic, Rita Bertani, Neda Lapteva, Panagis Drakatos, Alessio Delogu, Kausar Raheel, Matthew Soteriou, Carlotta Mutti, Joerg Steier, David W. Carmichael, Peter J. Goadsby, Adam Ockelford, Ivana Rosenzweig

**Affiliations:** ^1^Department of Neuroimaging, Sleep and Brain Plasticity Centre, Institute of Psychiatry, Psychology and Neuroscience, King’s College London, London, United Kingdom; ^2^BRAIN, Imaging Centre, CNS, King’s College London, London, United Kingdom; ^3^School of Basic and Medical Biosciences, Faculty of Life Sciences and Medicine, King’s College London, London, United Kingdom; ^4^Sleep Disorders Centre, Guy’s and St Thomas’ NHS Foundation Trust, London, United Kingdom; ^5^Department of Basic and Clinical Neuroscience, Institute of Psychiatry, Psychology and Neuroscience, King’s College London, London, United Kingdom; ^6^Department of Philosophy, King’s College London, London, United Kingdom; ^7^Department of General and Specialized Medicine, Sleep Disorders Center, University Hospital of Parma, Parma, Italy; ^8^Department of Biomedical Engineering, School of Biomedical Engineering and Imaging Sciences, King’s College London, London, United Kingdom; ^9^NIHR-Wellcome Trust King’s Clinical Research Facility, King’s College London, London, United Kingdom; ^10^Centre for Learning, Teaching and Human Development, School of Education, University of Roehampton, London, United Kingdom

**Keywords:** dreams, congenitally blind, cross-modal plasticity, metamodal brain, visuo-spatial imagery

## Abstract

**Background:**

The presence of visual imagery in dreams of congenitally blind people has long been a matter of substantial controversy. We set to systematically review body of published work on the presence and nature of oneiric visuo-spatial impressions in congenitally and early blind subjects across different areas of research, from experimental psychology, functional neuroimaging, sensory substitution, and sleep research.

**Methods:**

Relevant studies were identified using the following databases: EMBASE, MEDLINE and PsychINFO.

**Results:**

Studies using diverse imaging techniques and sensory substitution devices broadly suggest that the “blind” occipital cortex may be able to integrate non-visual sensory inputs, and thus possibly also generate visuo-spatial impressions. Visual impressions have also been reported by blind subjects who had near-death or out-of-body experiences.

**Conclusion:**

Deciphering the mechanistic nature of these visual impression could open new possibility in utilization of neuroplasticity and its potential role for treatment of neurodisability.

## 1. Introduction

The presence of visual imagery in dreams of congenitally blind people has long been a matter of substantial controversy (Berger et al., 1962; Amadeo and Gomez, 1966; Kirtley, 1975; Kerr et al., 1982; Hurovitz et al., 1999; Holzinger, 2000; Lopes Da Silva, 2003; Staunton and O’Rourke, 2012; Meaidi et al., 2014; Christensen et al., 2019; Andrade, 2021). However, several recent studies appear to support the notion that (oneiric) visuo-spatial imagery, whilst very rare, can occur during dreaming in congenitally blind people (Bértolo et al., 2003, 2017).

Interestingly, more recent research appears to support suggestion of significant neuroplasticity in congenitally blind, with 40% of the congenitally blind population reported to have an absolute musical pitch, compared to 0.01% of neurotypical population (Ockelford, 2021). Similarly, superior verbal memory (Amedi et al., 2003), and an enhanced auditory localisation have been shown for congenitally blind by comparison to normally sighted population (Lessard et al., 1998).

However, it remains unclear to what extent the absence of vision affects the overall development of multi-modal sensory sensitivity, and underlying all this, to what extent it impacts the development of the visual cortex and other brain regions. For instance, it is known that early brain development is dependent on inherent genetic code and *in utero* environment; neocortical functional realization is coded by genetic and molecular information, and independent on sensory input (O’Leary et al., 2007; Espinosa and Stryker, 2012). In primates and humans, primary visual cortex shows basic region-specific cytoarchitecture, organization of receptive fields and cortical columns, even in the absence of thalamic input (Hubel et al., 1976; Rakic, 1988; Rakic et al., 1991; Miyashita-Lin et al., 1999; Nakagawa et al., 1999). Nonetheless, the final cellular, molecular and synaptic profile does not develop without appropriate thalamic afferents (Rakic et al., 1991).

Thus, it appears that the visual input that triggers response from cortical neurons is necessary for maintenance, rather than development of primary visual cortex in humans (Espinosa and Stryker, 2012). Moreover, whilst total absence of visual experience has been shown to delay functional maturation of the striate cortex (Timney et al., 1978; Blakemore and Price, 1987; Fagiolini et al., 1994; Fernando Maya-Vetencourt and Origlia, 2012), it has been shown that different non-visual components of the environment may protect, and further moderate development of the visual system, even in the absence of the visual input (Fernando Maya-Vetencourt and Origlia, 2012). For example, in transgenic mice, overexpressing brain-derived neurotrophic factor in forebrain and reared in dark, primary visual area neurons responded normally to visual stimuli, suggesting that mice may develop a functional sight despite the lack of visual experience during the critical period (Gianfranceschi et al., 2003).

This is important as it implies that a lack of sensory stimulus in one system could be rescued by increased sensory-motor stimulation or additional stimulation of other sensory systems (environmental enrichment). For instance, in one study, an increased sensory-motor stimulation through exploratory behavior in an enriched environment has been shown to prevent effects of dark rearing on rodent visual cortex development (Bartoletti et al., 2004). This process has been long referred to as cross-modal plasticity (Fernando Maya-Vetencourt and Origlia, 2012).

Cross-modal plasticity is a cortical phenomenon when deprivation of one sensory input during critical period of development strengthens remaining sensory modalities (Fernando Maya-Vetencourt and Origlia, 2012). In keeping, several studies have shown that an enriched environment accelerates structural and functional development of rodent visual system (Cancedda et al., 2004; Fernando Maya-Vetencourt and Origlia, 2012). Similarly, it has been reported that body massage in human preterm infants increases IGF-1 serum levels and accelerates visual system maturation (Guzzetta et al., 2009; Fernando Maya-Vetencourt and Origlia, 2012). Taken together, this data indicates that increased stimulation of other sensory modalities accelerates development of visual cortex, even without visual deprivation. Consequently, this may suggest that any such visual system may contribute to oneiric visual imagery-alike perceptions even in blind.

Arguably, the creation of new connections between the occipital cortex and areas of the brain involved in auditory or haptic processing and/or the unmasking of existing connections, which are normally inhibited in the presence of vision (Bavelier and Neville, 2002; Burton, 2003; Müller et al., 2019), may, in the blind, enable integration of non-visual sensory inputs to generate any such visuo-spatial images. Another interesting possibility is that of human brain functioning as the intrinsically metamodal structure, organized as operators that execute a given function or computation regardless of sensory input modality [for more on this topic please also refer to Pascual-Leone and Hamilton (2001)].

Nonetheless, it remains unclear to what extent the absence of vision may affects the sensory sensitivity for oneiric construction and thus, in order to gain a better insight into possible sleep and neural mechanisms which may underlie this phenomenon, we set to systematically review and to critically analyze a body of published work on the presence and nature of oneiric visuo-spatial impressions in congenitally and early blind subjects across different areas of research, from experimental psychology, functional neuroimaging, sensory substitution, and sleep research. Finally, we hypothesized that (oneiric) mental representation of images may not be entirely dependent on visual input.

## 2. Materials and methods

### 2.1. Literature search

This systematic review was conducted following the Preferred Reporting Items for Systematic Reviews and Meta-Analyses (PRISMA) guidelines (Page et al., 2021). Relevant studies were identified using the online search databases EMBASE, MEDLINE and PsychINFO. The following keywords were used: {(exp *blindness/) AND [(exp dream/OR exp dreaming/) OR (exp REM sleep/) OR (*vision/OR exp visual hallucination/OR exp visual illusion/)]} in EMBASE, {(exp *Blindness/) AND [(exp Dreams/OR exp Sleep, REM/) OR [(Visual Hallucination* OR Visual Perception).mp]]} in MEDLINE, and {(exp *blind/) AND [(exp dreaming/OR exp dream analysis/) OR (exp rem sleep/OR exp rapid eye movement/OR exp rem dreams/) OR (exp visual hallucinations/OR *visual perception/)]} in PsychINFO. As each database employed different subject terms, different search string was chosen for each of the three databases (please see Table 1). Eligible studies were extracted from January 1960 to March 2021. The references of the selected studies were also examined to retrieve documents missed by the literature search.

**TABLE 1:** The search strategy and exclusion/inclusion criteria.

Database	Search strategy	Limits
EMBASE (Ovid)	{(exp *blindness/) AND [(exp dream/OR exp dreaming/) OR (exp REM sleep/) OR (*vision/OR exp visual hallucination/OR exp visual illusion/)]}	Year: 1960–2021 Species: human Only in English
MEDLINE (Ovid)	{(exp *blindness/) AND [(exp dreams/OR exp sleep, REM/) OR [(visual hallucination* OR visual perception).mp]]}	Year: 1960–2021 Species: human Only in English
PsychINFO	{(exp *blind/) AND [(exp dreaming/OR exp dream analysis/) OR (exp rem sleep/OR exp rapid eye movement/OR exp rem dreams/) OR (exp visual hallucinations/OR *visual perception/)]}	Year: 1960–2021 Species: human Only in English

### 2.2. Inclusion and exclusion criteria

**FIGURE 1:**
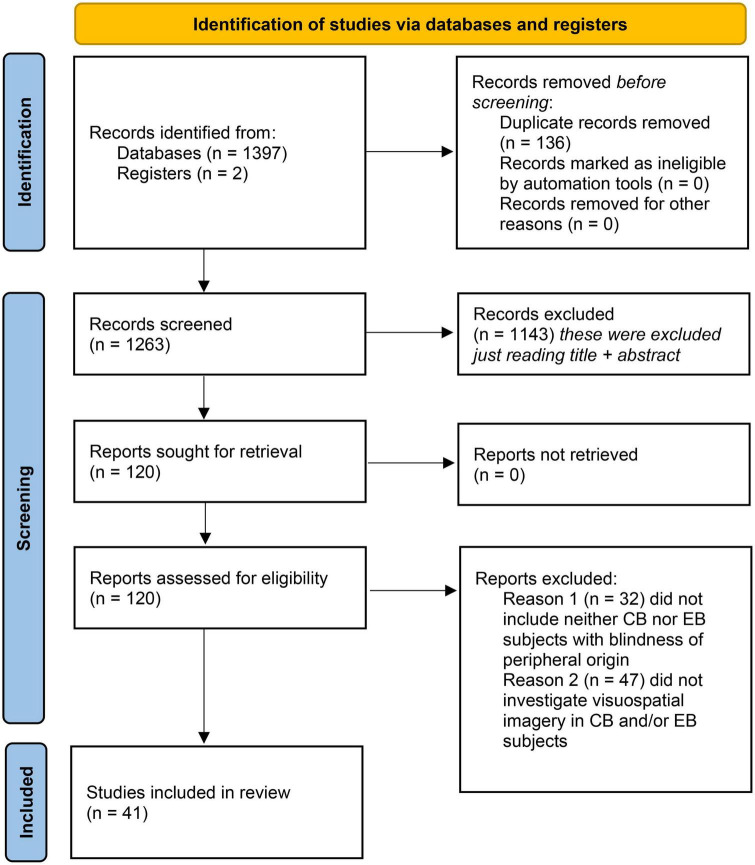
PRISMA 2020 flow diagram of study selection process (Liberati et al., 2009).

All databases were screened following the same protocol. The studies were included if they came from (1) original research articles published by a peer-reviewed scientific journal; (2) were written in English and (3) if they included observational, descriptive, longitudinal, retrospective, cross-sectional, or cohort studies. Included studies were required to investigate presence of visual imagery in congenitally blind participants, or early blind individuals with blindness of peripheral origin. Studies were excluded if they were case reports, or if they included only late blind participants. Two reviewers (NL and RB) independently screened each eligible study, and disagreements were resolved through discussion after retrieving full text to determine whether inclusion and exclusion criteria were met or consulting a third independent investigator (KI) (please also refer to Table 2). The PICOS statement is available in Table 3 and PRISMA 2020 flow diagram of study selection process is shown in Figure 1.

**TABLE 2:** The exclusion and inclusion criteria.

	Exclusion criteria	Inclusion criteria
Manuscript characteristics	Conference abstracts and proceedings, unpublished data, preprints, government publications and reports, dissertations, and theses Animal studies Studies involving under 18 s, infants, pediatric Guidelines, statements, and comments General review papers Meta-analyses, and systematic review	Original research articles Observational, descriptive, longitudinal, retrospective, cross-sectional, and cohort studies investigating the presence of visual imagery in congenitally blind or early blind individuals Sample was well-described (e.g., number of subjects, recruitment criteria, age mean or age range etc.)
Study design	Studies which included only late blind and/or partially sighted participants, or only participants with cortical “cerebral” blindness Studies in which neurotechnology was employed to assess navigation and motion perception in blind subjects were excluded, as well as studies of haptic perception only, or studies assessing memory for visual terms in blind versus sighted subjects (unless visuo-spatial imagery was directly investigated as part of the experiments)	Studies that are primary research investigating the presence of visual imagery, during sleep or wake in congenitally blind (individuals who were born blind) or early blind participants with blindness of peripheral origin (individuals who lost sight after 7 years of age) and/or controls (sighted subjects)

**TABLE 3:** The PICOS statement.

Component of question	Example
Population	Congenitally blind and/or early blind individuals
Intervention	None
Control	Sighted controls
Outcomes	Assessing the presence of visuospatial impressions in the imagery of congenitally and/or early blind subjects
Study design	Retrospective, longitudinal, observational, cohort studies, case-control studies, controlled trials

### 2.3. Data extraction

The following data were extracted: type of study, sample size, type of blindness, sex, age and handedness of participants; techniques and measures employed; main findings and study limitations (also please refer to Supplementary Tables 1–5). Subsequently, the studies were classified into five predetermined categories, based on the investigating techniques employed in the studies (i.e., neurotechnology, psychological investigations, sensory substitution or near-death and out-of-body experiences). A third independent investigator went through all extracted data and tables to reconcile any eventual disagreements.

## 3. Results

A total of 1,397 studies were identified through database searching (see Figure 1). Two more studies (Holzinger, 2000; Bértolo et al., 2017), cited in the literature but absent in the three databases used, were reviewed and added to the relevant studies, for a total of 1,399. Of remaining studies, only 41 studies were found to fulfill the exclusion and inclusion criteria (Figure 1) and they were consequently classified as belonging to one of the following predefined five categories, based on the techniques employed: (1) studies using neurotechnology to investigate the presence of visuo-spatial imagery in blind subjects during wake (10 studies; please refer for more in-depth analysis in the Supplementary Table 1); (2) psychological investigations of visuo-spatial imagery in blind subjects (11 studies; Supplementary Table 2); (3) studies investigating visuo-spatial imagery as a consequence of sensory substitution in blind subjects (11 studies; Supplementary Table 3); (4) studies investigating visuo-spatial imagery during near-death and out-of-body experiences in blind subjects (1 study); and finally (5) studies investigating the presence of visuo-spatial imagery in blind subjects’ dreams (8 studies; Supplementary Table 5). Of note is that many studies employed a combination of different techniques.

### 3.1. Studies using neurotechnology to investigate the presence of visuo-spatial imagery in blind subjects during wake

The changes in human brain in subjects that are either blind from birth or very early age (congenitally blind and early blind) and in those with blindness onset later in life (late blind) were investigated by several studies. Number of neuroimaging studies in blind report different patterns of activation in primary visual and associative cortexes, as well as in prefrontal cortex (Supplementary Table 1). Röder et al. (1997) used electroencephalography in congenitally blind and sighted control subjects and detected a marked slow negative potential over the occipital cortex of 15 congenitally blind subjects who were performing mental rotation and comparison of images explored haptically. This slow negative potential was not observed in sighted control subjects who performed the same tasks. In two similar studies by De Volder et al. (2001) and Vanlierde et al. (2003), positron emission tomography was used in early blind and sighted control subjects who were performing a visuo-spatial imagery task. In both subject groups, De Volder et al. (2001) observed activation in lateral occipito-temporal areas; Vanlierde et al. (2003) in the precuneus, superior parietal lobe and occipital gyrus. Early blind group demonstrated stronger occipital activations (Vanlierde et al., 2003), whilst activation of the right prefrontal cortex and left posterior cingulate gyrus was observed only in the sighted control group (De Volder et al., 2001).

Only two studies used functional magnetic resonance imaging to explore brain activity in blind in response to specific concepts (Striem-Amit et al., 2018; Mattioni et al., 2020). Mattioni et al. (2020) found that the ventral occipito-temporal cortex encodes different concepts in a similar way in early blind (to whom concepts were presented acoustically) and in sighted control subjects (to whom concepts were presented visually). However, in the other functional magnetic resonance imaging study (Striem-Amit et al., 2018), a difference was spotted between congenitally blind and sighted controls: while in both groups, abstract concepts (e.g., “freedom”) were correlated with activity in the lateral anterior temporal lobe, and concrete concepts (e.g., “cup”) with activity in medial anterior temporal lobe, concepts such as “red,” which are imperceptible for blind people, were correlated with activity in left dorsal anterior temporal lobe in the congenitally blind group only. In the same study by Striem-Amit et al. (2018), resting-state functional connectivity analysis revealed that, in both congenitally blind and sighted control, dorsal and lateral anterior temporal lobe are connected with areas involved in semantic, non-sensorially derived information (e.g., the inferior frontal lobe), while the medial anterior temporal lobe is connected to multisensory object-related regions in central visual cortex, and in frontal and parietal lobes.

### 3.2. Psychological investigations of visuo-spatial imagery in blind subjects

In the Supplementary Table 2, 11 studies are reported, in which mental representations of patterns, pathways, objects and spatial relations were compared between groups of congenitally blind, early blind, late blind and sighted control subjects. In three of those studies, blind subjects performed better or as well as sighted controls in visuo-spatial imagery tasks. For instance, after administering the Onomatopoeia and Images (Torrance et al., 1973), Johnson (1980) found that congenitally blind subjects produced more visual images than the sighted controls. Hollins (1985) reports that congenitally blind and sighted control subjects were equally good at identifying objects represented as patterns of squares on 2D matrices or of cubes on 3D matrices. Tinti et al. (2006) reported that both congenitally blind and late blind subjects were better than sighted controls at drawing two pathways they had just learned to walk through; however, when drawings of the two pathways were analyzed together, only late blind performed better than sighted controls.

In five of the eight remaining studies, blind and sighted subjects performed similarly well, although there were some interesting differences. For example, in one study (Kerr, 1983), the congenitally blind performed similarly to the sighted at imagining objects and their properties, although they struggled when they had to imagine concealed objects. Heller et al. (1996) found evidence of foreshortening in sighted control subjects only; however, drawings of a panel at different angular orientations were not significantly different between the early blind, the late blind and the sighted controls. In Vanlierde and Wanet-Defalque (2004), early blind, late blind and sighted control individuals performed similarly in a series of pattern recognition tasks, but they used different strategies: all 27 sighted controls, nine out of ten late blind and one out of ten early blind subjects used a “visual” strategy, while the remaining nine early blind subjects used a “coordinate” strategy, and the last late blind utilized a “mixed” (visual and coordinate) strategy. Noordzij et al. (2007) report that only their late blind–but not the early blind–group performed worse than sighted controls in a visual imagery task, while the early blind performed worse than the sighted in a spatial imagery task. In Cattaneo et al. (2010), both early blind and sighted controls participants remembered more symmetrical than non-symmetrical patterns; however, only in the sighted controls group vertical symmetries were recalled more easily than horizontal ones.

### 3.3. Studies investigating visuo-spatial imagery as a consequence of sensory substitution in blind subjects

Of the 11 sensory substitution studies selected for this review (Supplementary Table 3), five employed visual-to-tactile sensory substitution devices. Specifically, Miletic et al. (1988) used a modified version of the Optacon (Linvill et al., 1966), Sampaio et al. (2001) and Chebat et al. (2007) used the TDU (Bach-y-Rita et al., 1998), Velázquez and Bazán (2010) the “on-shoe tactile display” (Velázquez et al., 2009), while Nau et al. (2015) used the BrainPort Artificial Vision Device (Wicab Inc., Madison, WI, USA). In the remaining 6 studies, the authors employed a visual-to-auditory sensory substitution devices. Renier et al. (2005), Renier et al. (2006), and Renier and De Volder (2010) used the PSVA (Capelle et al., 1998), while Abboud et al. (2014), Buchs et al. (2015), and Buchs et al. (2019) employed EyeMusic, an sensory substitution devices which uses different musical instruments to convey color information (Abboud et al., 2014).

### 3.4. Studies investigating visuo-spatial imagery during near-death and out-of-body experiences in blind subjects

Ring and Cooper (1997) interviewed 14 early blind, 11 late blind and 6 visually impaired subjects who had at least one near-death experience or one out-of-body experience, and found that 25 (80%) of their 31 subjects–9 (64%) out of 14 early blind subjects–claimed sight during near-death experiences and/or out-of-body experiences. These visual experiences included seeing one’s own physical body, going through a tunnel or dark space, and seeing a radiant light. Interestingly, when asked to compare near-death experiences and out-of-body experiences with their usual dream content, early blind individuals answered that visual experiences were present only in their near-death experiences/out-of-body experiences.

### 3.5. Studies investigating the presence of visuo-spatial imagery in blind subjects’ dreams

**FIGURE 2:**
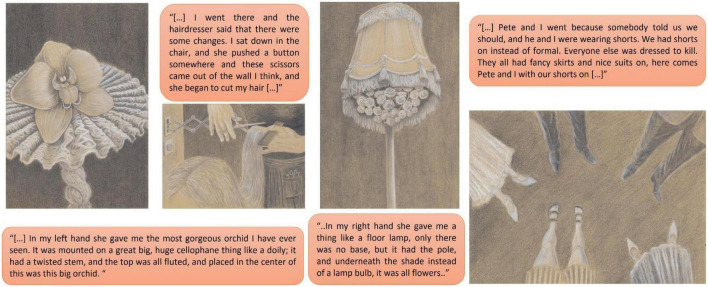
Representative dreams from the group of congenitally blind subjects, with examples of reported visuo-spatial imagery, adapted from with permission from Kang et al. (2023) (Illustrations by Davor Aslanovski).

Several studies have demonstrated that congenitally blind people can experience oneiric visuo-spatial imagery in a way similar to sighted individuals (see Figure 2 and Supplementary Table 5; Bértolo et al., 2003, 2017; Bértolo, 2005). Moreover, some congenitally blind subjects have also been able to represent the visual content of their dreams in drawings which were similarly accurate, if somewhat less detailed, and slightly more symbolic and archetypal, as those of sighted controls (Bértolo et al., 2003). Also, in keeping with positive findings by Bértolo et al. (2003, 2017) and Bértolo (2005), some of the past studies concomitantly report findings at odds with their own negative conclusion that congenitally, and early blind subjects completely lack oneiric visual imagery. For instance, in Hurovitz et al. (1999), one of the early blind participants, totally blind since the age of 4, reported “not metaphorical” visual imagery in his dream reports; in Christensen et al. (2019), visual dream elements were reported by congenitally blind subjects on two occasions; Meaidi et al. (2014) write that “many of the blind participants in our study described an object or a scene verbally in such rich visual terms that the interlocutor began to doubt if these individuals really lacked vision.”

## 4. Discussion

In this systematic review, we analyzed 41 studies on the presence and nature of visuo-spatial imagery in the blind, with the overarching goal of elucidating what happens in the visual cortex of blind people. We set out to investigate how blind people “see,” whether they may recreate visuo-spatial imagery via sensory substitution and, finally, whether they are able to dream in images.

### 4.1. What happens in the visual cortex of blind people?

Whilst numerous studies in animals have elucidated how the visual cortex is developed, and how it changes in cases of monocular or binocular deprivation during different periods after birth, relative sparsity of human studies and technologic limitations have so far prevented us from gaining similar insight into human development. A complex picture emerges from reported studies: during visuo-spatial imagery tasks, the occipital cortex is activated in the blind more than in the sighted (Röder et al., 1997; Arno et al., 2001; Vanlierde et al., 2003; Collignon et al., 2007; Striem-Amit et al., 2012; Striem-Amit and Amedi, 2014), and possibly in substitution for prefrontal activity, which was sometimes detected in the sighted but not in the blind (Arno et al., 2001). However, in the occipital, temporal and parietal lobes of blind subjects, higher-order areas, which are involved in multisensory integration and supramodal representations, seem to generally preserve their function and connectivity (Arno et al., 2001; Vanlierde et al., 2003; Amedi et al., 2007; Striem-Amit and Amedi, 2014; Striem-Amit et al., 2018; Mattioni et al., 2020). Nonetheless, several distinctive features have been recorded in the blind; for instance, representations of concrete concepts that are perceptible by the sighted but not by the blind have been shown to involve activations in the left dorsal anterior temporal lobe in blind subjects only (Striem-Amit et al., 2018).

### 4.2. How does a blind brain see?

In blind subjects, the occipital cortex is activated during haptic (Amedi et al., 2010) and auditory (Kujala et al., 1995, 2005; Liotti et al., 1998; Weeks et al., 2000) processing, including pitch memory tasks (Gaab et al., 2006); “visual” cortex activation in the blind has been associated with episodic retrieval (Raz et al., 2005) and with Braille reading (Sadato et al., 1996); transcranial magnetic stimulation (TMS) used to disrupt occipital activity leads to errors during Braille reading in blind subjects, but not in sighted people (Cohen et al., 1997). Moreover, when used to stimulate occipital areas, TMS induces subjective sensations (qualia) in the fingertips of blind subjects, and then in the tongue after training with the sensory substitution device TDU (Kupers et al., 2006).

This is of note, as the geniculo-striate system is atrophic in the blind, with up to 25% volume decrease in the BA17 cortical region (Ptito et al., 2008). The creation of new corticocortical and/or thalamocortical connections, or the unmasking of pre-existing connections, normally inhibited in the presence of vision, has been suggested as a potential mechanism behind the involvement of the “blind” visual cortex in non-visual sensory processing (Sadato et al., 1996; Cohen et al., 1997; Liotti et al., 1998; Arno et al., 2001; Burton, 2003; Kupers et al., 2006, 2011; Müller et al., 2019). Indeed, in animals, such as for example blind moles, the inferior colliculus projects not only to the auditory medial geniculate nucleus, but also to the visual lateral geniculate nucleus of the thalamus (Doron and Wollberg, 1994). In humans, diffusion tensor imaging revealed microstructural changes within thalamic clusters in blind individuals, even when no gross differences in the thalamocortical network were observed (Reislev et al., 2017). In keeping, Müller et al. (2019) have proposed the existence of a thalamocortical pathway for fast rerouting of haptic information to the blind “visual” cortex, activated only 35 ms after haptic stimulation in congenitally blind subjects, but not in blindfolded sighted people.

Different strategies in perceiving objects by sighted and blind were previously argued by Révész and Wolff (1950) in *Psychology and Art of the Blind*. He distinguished between “Haptics” and “Optics,” which, in his opinion, lead to different representations of objects, shapes and space in blind and sighted subjects. He argued that an object can be immediately perceived in its entirety through vision, while it can only be gradually “discovered” in a series of subsequent haptic explorations by a blind person. Thus, according to Révész and Wolff (1950), the same object must be represented in qualitatively different ways between the blind and sighted individuals. Indeed, considering diverging findings from analyzed studies, it remains unclear which mechanistic processes may support the presence and nature of visuo-spatial imagery in blind individuals. It is obvious that congenitally blind and early blind subjects may have some mental representation of patterns, shapes and spatial relations. Nonetheless, it is not clear how much these representations are affected by the blind’s main perceptual modality (i.e., haptics), and therefore how much visuo-spatial representations in the blind differ from those of the sighted. For instance, it appears that while congenitally blind and early blind subjects are as good as–or even better than–sighted controls at drawing pathways, imagining objects and remembering 2D patterns, they struggle to imagine concealed objects and reproduce 3D patterns, and they lack foreshortening.

### 4.3. Can we produce visuo-spatial imagery via sensory substitution in blind subjects?

A century ago, Romains (1920) managed to convey visuo-spatial information through the skin of blindfolded sighted subjects. Later in the 20th century, Starkiewicz and Kuliszewski (1963) developed the “Elektroftalm,” a device which transformed visuo-spatial images into patterns of vibrations, enabling blind subjects to “see.” Another pioneering method of sensory substitution was trialed by Bach-y-Rita et al. (1969), whose research group built an sensory substitution device consisting of a camera mounted on a dental chair. Here, visual information from the camera was converted to vibratory stimulation of the back of blind people sitting on the chair (Bach-y-Rita et al., 1969). Since then, the field of sensory substitution has grown and seen the development of various sensory substitution devices, not only for the blind; see for example Riso (1999) or Tyler et al. (2003).

In this review, we included 11 recent studies in which visual-to-auditory or visual-to-vibrotactile sensory substitution devices were employed to investigate visuo-spatial perception and imagery in congenitally and/or early blind subjects. Overall, it would appear that congenitally blind, early blind and late blind, as well as sighted subjects, can integrate non-visual information (auditory and haptic in this case) to create visuo-spatial mental images. However, there are differences between vision as a result of visual inputs and “blind vision” acquired through sensory substitution. For example, blind subjects using sensory substitution devices were shown insensitive to either Ponzo (Renier et al., 2005) or vertical-horizontal (Renier et al., 2006) illusions, and were similarly shown to have difficulties recognizing 3D shapes and spatial relations (Miletic et al., 1988; Velázquez and Bazán, 2010). Thus, one could argue that whilst visual imagery is possible even in the absence of inputs from the retina, it is still influenced by the specific sensory modality in which information is conveyed to visual association areas.

### 4.4. Visual experience from the beyond

Anecdotally, visuo-spatial imagery has been reported during near-death and out-of-body experiences by the blind, albeit the mechanistic nature of any such imagery remains poorly understood (Ring and Cooper, 1997). Near-death or out-of-body experience are rare parapsychological phenomena reported to occur during clinical cardio-respiratory arrest, or other drug or accident induced states, when a person seems to be awake and sees his body and the world from a location outside his physical body (Bünning and Blanke, 2005) [for an in-depth overview of the topic, also see *Mindsight: Near-Death and Out-of-Body Experiences in the Blind* (Ring and Cooper, 1999)].

### 4.5. Do congenitally blind people dream in images?

The above body of data may be taken to support the notion that (oneiric) mental representation of images may not be dependent on visual input, and that they can therefore be a, (albeit infrequent) part of congenitally blind individuals’ dreams.

Past evidence suggests that congenitally blind people may experience more circadian dysregulation (Lockley et al., 2007) and sleep-related problems (64% of congenitally blind subjects) than late blind or sighted controls (43 and 30%, respectively), as assessed using the Pittsburgh Sleep Quality Index. They also report suffering with more nightmares [mean frequency of nightmares over total number of dream reports in congenitally blind subjects: (25%) than late blind (7%) or sighted controls (6%) (Meaidi et al., 2014)].

However, the presence of visual imagery in the oneiric mentation in congenitally blind remains widely disputed, and highly debatable, despite some anecdotal, as well as some limited scientific evidence to the contrary (Berger et al., 1962; Amadeo and Gomez, 1966; Kirtley, 1975; Kerr et al., 1982; Hurovitz et al., 1999; Holzinger, 2000; Lopes Da Silva, 2003; Staunton and O’Rourke, 2012; Meaidi et al., 2014; Christensen et al., 2019; Andrade, 2021). It is also possible, that any such reports might have been historically ignored and disregarded (Kerr et al., 1982; Hurovitz et al., 1999; Holzinger, 2000; Staunton and O’Rourke, 2012; Meaidi et al., 2014; Christensen et al., 2019). Instead, it has been widely accepted that auditory, haptic, proprioceptive, somaesthetic, olfactory and gustatory imagery, as well as that of pain and temperature may be more prevalent in dreams of congenitally blind, by comparison to sighted people (Hurovitz et al., 1999; Holzinger, 2000; Staunton and O’Rourke, 2012; Meaidi et al., 2014; Supplementary Table 5).

When analyzing dream contents, most studies failed to record reports of visual impressions in the dreams of congenitally blind individuals (Kerr et al., 1982; Hurovitz et al., 1999; Holzinger, 2000; Staunton and O’Rourke, 2012; Meaidi et al., 2014), with only visual impressions (color, light) reported in the dreams of congenitally blind subjects with residual vision (Kerr et al., 1982; Meaidi et al., 2014). In late blind participants, the average blindness duration index has been shown to negatively correlates with duration, clarity, and color content of visual impressions in dreams (Meaidi et al., 2014). Positive correlations between lack of oneiric visual imagery and (1) congenital or early blindness, (2) total blindness, and (3) a high percentage of the subject’s life spent being blind has been reported by Hurovitz et al. (1999). Interestingly, some late blind subjects reported experiencing oneiric visual imagery (including colors) of people and objects only after the onset of blindness (Kerr et al., 1982; Holzinger, 2000). In that respect, it is of note that Kirtley (1975) proposed that, if the onset of blindness is before the age of five, no visual experience in the dreams should be possible, whilst onset of blindness between the ages five and seven may produce some oneiric visual imagery. It has been thus proposed that visual imagery in dreams will be retained only if onset of blindness happens after age of seven. Conversely, in one study by Hurovitz et al. (1999) it has been suggested that oneiric visual impressions may be retained if sight was lost after the age of four. Moreover, Meaidi et al. (2014) argued that only individuals who were born blind (congenitally blind), or who lost their sight before 2.5 years of age, will completely lack visual imagery in their dreams.

In this background, it is also of note that congenitally blind people have been shown to have significantly reduced, or absent, rapid eye movements (REM) during sleep (Christensen et al., 2019), whilst they appear to retain some of the other EEG features of phasic REM sleep microstructure. The phasic periods of REM sleep are commonly discerned by bursts of eye movements linked to so-called ponto-geniculo-occipital waves, contractions of the middle ear muscles, myoclonic twitches of skeletal muscles, sawtooth waves, as well as irregularities in cardio-respiratory activity (Simor et al., 2020). Some authors have suggested that the phasic REM sleep may play its own specific role in dreaming and emotion regulation processes during sleep, likely relating to reactivation of vivid visuo-spatial (emotionally relevant) mental pictures, or to the reprocessing of emotional memories during sleep (Simor et al., 2020). Thus, arguably, in blind, differential neurocircuitry may be activated during the phasic REM to that in sighted individuals, possibly reflecting use of auditory (or haptic), instead of visual referencing points. However, it remains unclear whether this may also translate in differential functional role of this fundamental physiologic process.

This is of particular importance given that, sleep itself, and more specifically, REM sleep (Marks et al., 1995) is crucial for the neurodevelopment of the visual cortex (Frank et al., 2001; Hobson, 2009), and therefore of mental imagery (Foulkes, 1982, 1999; Hobson, 2009; Siclari et al., 2020).

## 5. Conclusion, congenitally blind and the phenomenon of cross-modal neuroplasticity

At the neurophysiologic level, it would appear that the existence of visual-alike mental imagery could be argued by the demonstrations of cross-modal neuroplasticity, as evidenced by the neuroimaging (Röder et al., 1997; Arno et al., 2001; Vanlierde et al., 2003) and sensory substitution (Bach-y-Rita et al., 1969; Abboud et al., 2014; Buchs et al., 2019) studies. For instance, as previously discussed, studies using sensory substitution devices demonstrate that congenitally blind and early blind individuals can use auditory or haptic inputs to generate visuo-spatial representations of the world around them. These representations are accompanied by activation of brain areas such as extrastriate body area and lateral-occipital tactile-visual area, as demonstrated by neuroimaging studies. In the blind, activation of these and similar areas during visuo-spatial imagery tasks suggests that visual perception is not necessary for the creation of supramodal representations through multisensory integration. The visual system consists of complex parallel and interacting processing pathways in the brain which process neural information on form, motion, and color (Hasson et al., 2002; Bértolo, 2005). However, even for a neurotypical visual system, it remains ambiguous how separate pathways of visual system are brought together into a single image, and perhaps even more pertinently, whether mental imagery activated all of them (Hasson et al., 2002; Bértolo, 2005; Pulvermuller, 2018).

Arguably, if present, any such phenomenon of the cross-modal plasticity of the blind occipital cortex could be argued to support cortical deference (i.e., the thesis that the function of a specific brain area is determined by the type of input received) over cortical dominance (i.e., the idea that brain areas are pre-determined in their function, independently of sensory inputs) (Hurley and Noë, 2003). Moreover, this notion would then also support the idea that the “visual” cortex of the blind is anything but visual. Nonetheless, there are some valid arguments against this notion. For instance, if this was the case, this would not fully explain why the occipital cortex of congenitally blind people maintains the division between ventral (shape-processing) and dorsal (location-processing) pathways, normally found in the sighted (Striem-Amit et al., 2012). Moreover, several fundamental neurophysiologic processes and structure of sleep remain surprisingly intact in congenitally blind people (Bértolo et al., 2003). In addition, Aubin et al. (2018) report decreased alpha power and a generally higher resting-state metabolic rate over the occipital cortex during wakefulness, as well as REM sleep, in blind subjects. This constant increase of occipital activity in the blind, compared to the sighted individuals, has been linked to multisensory integration and therefore could underlie process of visual imagery in blind. Conversely, it could also represent an involvement of the blind “visual” cortex in higher-order functions–such as episodic memory (Raz et al., 2005) and language processing (Röder et al., 2000)–which are not directly related to the creation of visuo-spatial images.

More recently, it has been also shown that circuitry underlying REM sleep serves to selectively amplify the visual system’s activity periodically throughout the night, possibly preventing unregulated neuroplastic changes and takeover from other sensory inputs (Eagleman and Vaughn, 2021). It would follow that, during REM dreaming in blind, therefore, the impressions generated by different sensory modalities (e.g., auditory, haptic) can be extracted and integrated into a richer visual imagery-alike percept, in part thanks to the eccentric genetic wiring of our early visual cortex (Vetter et al., 2020). In turn, this may then enable development of a typical spatio-temporal organization of early visual areas by eccentricity (Hasson et al., 2002), even in the life-long absence of vision (Eagleman and Vaughn, 2021). Finally, this could also explain ability of some congenitally blind individuals to draw symbolic representations of various visual images (Bértolo et al., 2017) in striking likeness to those drawn by normally sighted.

## Author contributions

KI and RB selected and reviewed the studies. KI, NL, and RB assessed all the eligible studies. All authors involved in reviewing and drafting the manuscript and approved the submitted version.

## References

[B1] Abboud S. Hanassy S. Levy-Tzedek S. Maidenbaum S. Amedi A. (2014). EyeMusic: Introducing a “visual” colorful experience for the blind using auditory sensory substitution. *Restor. Neurol. Neurosci.* 32 247–257. 10.3233/RNN-130338 24398719

[B2] Amadeo M. Gomez E. (1966). Eye Movements, Attention and Dreaming in Subjects with Lifelong Blindness. *Can. Psychiatric Assoc. J.* 11 501–507. 10.1111/jsr.12866 31025801

[B3] Amedi A. Raz N. Azulay H. Malach R. Zohary E. (2010). Cortical activity during tactile exploration of objects in blind and sighted humans. *Restor. Neurol. Neurosci.* 28 143–156. 10.3233/RNN-2010-0503 20404404

[B4] Amedi A. Raz N. Pianka P. Malach R. Zohary E. (2003). Early “visual” cortex activation correlates with superior verbal memory performance in the blind. *Nat. Neurosci.* 6 758–766. 10.1038/nn1072 12808458

[B5] Amedi A. Stern W. M. Camprodon J. A. Bermpohl F. Merabet L. Rotman S. (2007). Shape conveyed by visual-to-auditory sensory substitution activates the lateral occipital complex. *Nat. Neurosci.* 10 687–689. 10.1038/nn1912 17515898

[B6] Andrade M. J. O. (2021). Do congenitally blind people have visual dreams? *Sleep Sci.* 14 190–192. 10.5935/1984-0063.20200068 34381585 PMC8340899

[B7] Arno P. De Volder A. G. Vanlierde A. Wanet-Defalque M.-C. Streel E. Robert A. (2001). Occipital activation by pattern recognition in the early blind using auditory substitution for vision. *NeuroImage* 13 632–645. 11305892 10.1006/nimg.2000.0731

[B8] Aubin S. Christensen J. A. E. Jennum P. Nielsen T. Kupers R. Ptito M. (2018). Preserved sleep microstructure in blind individuals. *Sleep Med.* 42 21–30. 10.1016/j.sleep.2017.11.1135 29458742

[B9] Bach-y-Rita P. Collins C. C. Saunders F. A. White B. Scadden L. (1969). Vision substitution by tactile image projection. *Nature* 221 963–964. 10.1038/221963a0 5818337

[B10] Bach-y-Rita P. Kaczmarek K. A. Tyler M. E. Garcia-Lara J. (1998). Form perception with a 49-point electrotactile stimulus array on the tongue: a technical note. *J. Rehabil. Res. Dev.* 35 427–430. 10220221

[B11] Bartoletti A. Medini P. Berardi N. Maffei L. (2004). Environmental enrichment prevents effects of dark-rearing in the rat visual cortex. *Nat. Neurosci.* 7 215–216. 14966527 10.1038/nn1201

[B12] Bavelier D. Neville H. J. (2002). Cross-modal plasticity: where and how? *Nat. Rev. Neurosci.* 3 443–452. 10.1038/nrn848 12042879

[B13] Berger R. J. Olley P. Oswald I. (1962). The EEG, eye-movements and dreams of the blind. *Q. J. Exp. Psychol.* 14 183–186. 10.1097/00005053-196509000-00014 5846596

[B14] Bértolo H. (2005). Visual imagery without visual perception. *Psicologica* 26 173–188. 10.1016/j.cortex.2020.11.014 33383478 PMC7856239

[B15] Bértolo H. Mestre T. Barrio A. Antona B. (2017). *Rapid Eye Movements (REMs) and visual dream recall in both congenitally blind and sighted subjects.* Paris: SPIE.

[B16] Bértolo H. Paiva T. Pessoa L. Mestre T. Marques R. Santos R. (2003). Visual dream content, graphical representation and EEG alpha activity in congenitally blind subjects. *Brain Res. Cogn. Brain Res.* 15 277–284. 10.1016/s0926-6410(02)00199-4 12527101

[B17] Blakemore C. Price D. J. (1987). Effects of dark-rearing on the development of area 18 of the cat’s visual cortex. *J. Physiol.* 384 293–309. 10.1113/jphysiol.1987.sp016455 3656148 PMC1192263

[B18] Buchs G. Heimler B. Amedi A. (2019). The effect of irrelevant environmental noise on the performance of visual-to-auditory sensory substitution devices used by blind adults. *Multisens. Res.* 32 87–109. 10.1163/22134808-20181327 31059468

[B19] Buchs G. Maidenbaum S. Levy-Tzedek S. Amedi A. (2015). Integration and binding in rehabilitative sensory substitution: Increasing resolution using a new Zooming-in approach. *Restor. Neurol. Neurosci.* 34 97–105. 10.3233/RNN-150592 26518671 PMC4927841

[B20] Bünning S. Blanke O. (2005). The out-of body experience: precipitating factors and neural correlates. *Progress Brain Res.* 150 331–606. 10.1016/S0079-6123(05)50024-4 16186034

[B21] Burton H. (2003). Visual cortex activity in early and late blind people. *J. Neurosci.* 23:4005. 10.1523/JNEUROSCI.23-10-04005.2003 12764085 PMC3667661

[B22] Cancedda L. Putignano E. Sale A. Viegi A. Berardi N. Maffei L. (2004). Acceleration of visual system development by environmental enrichment. *J. Neurosci.* 24 4840–4848. 10.1523/JNEUROSCI.0845-04.2004 15152044 PMC6729456

[B23] Capelle C. Trullemans C. Arno P. Veraart C. (1998). A real-time experimental prototype for enhancement of vision rehabilitation using auditory substitution. *IEEE Trans. Biomed. Eng.* 45 1279–1293. 10.1109/10.720206 9775542

[B24] Cattaneo Z. Fantino M. Silvanto J. Tinti C. Pascual-Leone A. Vecchi T. (2010). Symmetry perception in the blind. *Acta Psychol.* 134 398–402. 10.1016/j.actpsy.2010.04.002 20444438

[B25] Chebat D. R. Rainville C. Kupers R. Ptito M. (2007). Tactile-“visual” acuity of the tongue in early blind individuals. *Neuroreport* 18 1901–1904. 10.1097/WNR.0b013e3282f2a63 18007183

[B26] Christensen J. A. E. Aubin S. Nielsen T. Ptito M. Kupers R. Jennum P. (2019). Rapid eye movements are reduced in blind individuals. *J. Sleep Res.* 28:e12866. 10.1111/jsr.12866 31025801

[B27] Cohen L. G. Celnik P. Pascual-Leone A. Corwell B. Faiz L. Dambrosia J. (1997). Functional relevance of cross-modal plasticity in blind humans. *Nature* 389 180–183. 10.1038/38278 9296495

[B28] Collignon O. Lassonde M. Lepore F. Bastien D. Veraart C. (2007). Functional cerebral reorganization for auditory spatial processing and auditory substitution of vision in early blind subjects. *Cereb. Cortex* 17 457–465. 10.1093/cercor/bhj162 16581983

[B29] De Volder A. G. Toyama H. Kimura Y. Kiyosawa M. Nakano H. Vanlierde A. (2001). Auditory triggered mental imagery of shape involves visual association areas in early blind humans. *Neuroimage* 14 129–139. 10.1006/nimg.2001.0782 11525322

[B30] Doron N. Wollberg Z. (1994). Cross-modal neuroplasticity in the blind mole rat Spalax ehrenbergi: a WGA-HRP tracing study. *Neuroreport* 5 2697–2701. 10.1097/00001756-199412000-00072 7535122

[B31] Eagleman D. M. Vaughn D. A. (2021). The defensive activation theory: REM sleep as a mechanism to prevent takeover of the visual cortex. *Front. Neurosci.* 15:632853. 10.3389/fnins.2021.632853 34093109 PMC8176926

[B32] Espinosa J. S. Stryker M. P. (2012). Development and plasticity of the primary visual cortex. *Neuron* 75 230–249. 10.1016/j.neuron.2012.06.009 22841309 PMC3612584

[B33] Fagiolini M. Pizzorusso T. Berardi N. Domenici L. Maffei L. (1994). Functional postnatal development of the rat primary visual cortex and the role of visual experience: dark rearing and monocular deprivation. *Vis. Res.* 34 709–720. 8160387 10.1016/0042-6989(94)90210-0

[B34] Fernando Maya-Vetencourt J. Origlia N. (2012). Visual cortex plasticity: A complex interplay of genetic and environmental influences. *Neural Plasticity* 2012:631965. 10.1155/2012/631965 22852098 PMC3407658

[B35] Foulkes D. (1982). *Children’s dreams: longitudinal studies.* New York, NY: Wiley.

[B36] Foulkes D. (1999). *Children’s Dreaming and the Development of Consciousness.* Cambridge, MA: Harvard University Press.

[B37] Frank M. G. Issa N. P. Stryker M. P. (2001). Sleep Enhances Plasticity in the Developing Visual Cortex. *Neuron* 30 275–287. 10.1016/s0896-6273(01)00279-3 11343661

[B38] Gaab N. Schulze K. Ozdemir E. Schlaug G. (2006). Neural correlates of absolute pitch differ between blind and sighted musicians. *Neuroreport* 17 1853–1857. 10.1097/WNR.0b013e3280107bee 17179857

[B39] Gianfranceschi L. Siciliano R. Walls J. Morales B. Kirkwood A. Huang Z. J. (2003). Visual cortex is rescued from the effects of dark rearing by overexpression of BDNF. *Proc. Natl. Acad. Sci. U. S. A.* 100 12486–12491. 10.1073/pnas.1934836100 14514885 PMC218784

[B40] Guzzetta A. Baldini S. Bancale A. Baroncelli L. Ciucci F. Ghirri P. (2009). Massage accelerates brain development and the maturation of visual function. *J. Neurosci.* 29 6042–6051. 19420271 10.1523/JNEUROSCI.5548-08.2009 PMC6665233

[B41] Hasson U. Levy I. Behrmann M. Hendler T. Malach R. (2002). Eccentricity bias as an organizing principle for human high-order object areas. *Neuron* 34 479–490. 11988177 10.1016/s0896-6273(02)00662-1

[B42] Heller M. A. Calcaterra J. A. Tyler L. A. Burson L. L. (1996). Production and interpretation of perspective drawings by blind and sighted people. *Perception* 25 321–334. 8804095 10.1068/p250321

[B43] Hobson J. A. (2009). REM sleep and dreaming: towards a theory of protoconsciousness. *Nat. Rev. Neurosci.* 10 803–813. 10.1038/nrn2716 19794431

[B44] Hollins M. (1985). Styles of mental imagery in blind adults. *Neuropsychologia* 23 561–566. 10.1016/0028-3932(85)90009-0 4033908

[B45] Holzinger B. (2000). The Dreams of the Blind: In Consideration of the Congenital and Adventitiously Blind. *J. Sleep Res.* 9:83.

[B46] Hubel D. Wiesel T. LeVay S. (1976). Functional architecture of area 17 in normal and monocularly deprived macaque monkeys. *Cold Spring Harb. Symp. Quant. Biol.* 40 581–589. 10.1101/sqb.1976.040.01.054 820507

[B47] Hurley S. Noë A. (2003). Neural Plasticity and Consciousness. *Biol. Philos.* 18 131–168.

[B48] Hurovitz C. S. Dunn S. Domhoff G. W. Fiss H. (1999). The dreams of blind men and women: A replication and extension of previous findings. *Dreaming* 9 183–193.

[B49] Johnson R. A. (1980). Sensory images in the absence of sight: blind versus sighted adolescents. *Percept. Mot. Skills* 51 177–178. 10.2466/pms.1980.51.1.177 7432954

[B50] Kang J. Bertani R. Raheel K. Soteriou M. Rosenzweig J. Valentin A. (2023). Homoiōma in Dreams of Congenitally Blind. *Sci. Rep.* 10.21203/rs.3.rs-1614543/v1 [Epub ahead of print]. PMC10605848 37891763

[B51] Kerr N. H. (1983). The role of vision in “visual imagery” experiments: evidence from the congenitally blind. *J. Exp. Psychol. Gen.* 112 265–277. 10.1037//0096-3445.112.2.265 6223973

[B52] Kerr N. H. Foulkes D. Schmidt M. (1982). The structure of laboratory dream reports in blind and sighted subjects. *J. Nerv. Mental Dis.* 170 286–294. 10.1097/00005053-198205000-00006 7069416

[B53] Kirtley D. D. (1975). *The psychology of blindness.* Oxford: Nelson-Hall.

[B54] Kujala T. Huotilainen M. Sinkkonen J. Ahonen A. I. Alho K. Hämälä:inen M. S. (1995). Visual cortex activation in blind humans during sound discrimination. *Neurosci. Lett.* 183 143–146. 7746476 10.1016/0304-3940(94)11135-6

[B55] Kujala T. Palva M. J. Salonen O. Alku P. Huotilainen M. Järvinen A. (2005). The role of blind humans’ visual cortex in auditory change detection. *Neurosci. Lett.* 379 127–131. 10.1016/j.neulet.2004.12.070 15823429

[B56] Kupers R. Fumal A. de Noordhout A. M. Gjedde A. Schoenen J. Ptito M. (2006). Transcranial magnetic stimulation of the visual cortex induces somatotopically organized qualia in blind subjects. *Proc. Natl. Acad. Sci.* 103:13256. 10.1073/pnas.0602925103 16916936 PMC1550769

[B57] Kupers R. Pietrini P. Ricciardi E. Ptito M. (2011). The Nature of Consciousness in the Visually Deprived Brain. *Front. Psychol.* 2:19. 10.3389/fpsyg.2011.00019 21713178 PMC3111253

[B58] Lessard N. Paré M. Lepore F. Lassonde M. (1998). Early-blind human subjects localize sound sources better than sighted subjects. *Nature* 395 278–280. 10.1038/26228 9751055

[B59] Liberati A. Altman D. G. Tetzlaff J. Mulrow C. Gøtzsche P. C. Ioannidis J. P. A. (2009). The PRISMA statement for reporting systematic reviews and meta-analyses of studies that evaluate healthcare interventions: explanation and elaboration. *BMJ* 339 b2700. 10.1136/bmj.b2700 19622552 PMC2714672

[B60] Linvill J. G. Bliss J. C. direct A. (1966). translation reading aid for the blind. *Proc. IEEE* 54 40–51.

[B61] Liotti M. Ryder K. Woldorff M. G. (1998). Auditory attention in the congenitally blind: Where, when and what gets reorganized? *Neuroreport* 9 1007–1012. 10.1097/00001756-199804200-00010 9601658

[B62] Lockley S. W. Arendt J. Skene D. J. (2007). Visual impairment and circadian rhythm disorders. *Dialogues Clin. Neurosci.* 9 301–314. 17969867 10.31887/DCNS.2007.9.3/slockley PMC3202494

[B63] Lopes Da Silva F. H. (2003). Visual dreams in the congenitally blind? *Trends Cogn. Sci.* 7 328–330. 10.1016/s1364-6613(03)00155-4 12907221

[B64] Marks G. A. Shaffery J. P. Oksenberg A. Speciale S. G. Roffwarg H. P. (1995). A functional role for REM sleep in brain maturation. *Behav. Brain Res.* 69 1–11. 10.1016/0166-4328(95)00018-o 7546299

[B65] Mattioni S. Rezk M. Battal C. Bottini R. Mendoza K. E. C. Oosterhof N. N. (2020). Categorical representation from sound and sight in the ventral occipito-temporal cortex of sighted and blind. *eLife* 9:e50732. 10.7554/eLife.50732 PMC7108866 32108572

[B66] Meaidi A. Jennum P. Ptito M. Kupers R. (2014). The sensory construction of dreams and nightmare frequency in congenitally blind and late blind individuals. *Sleep Med.* 15 586–595. 10.1016/j.sleep.2013.12.008 24709309

[B67] Miletic G. Hughes B. Bach-y-Rita P. (1988). Vibrotactile stimulation: An educational program for spatial concept development. *J. Vis. Impair. Blind.* 82 366–370.

[B68] Miyashita-Lin E. M. Hevner R. Wassarman K. M. Martinez S. Rubenstein J. L. (1999). Early neocortical regionalization in the absence of thalamic innervation. *Science* 285 906–909. 10.1126/science.285.5429.906 10436162

[B69] Müller F. Niso G. Samiee S. Ptito M. Baillet S. Kupers R. (2019). A thalamocortical pathway for fast rerouting of tactile information to occipital cortex in congenital blindness. *Nat. Commun.* 10:5154. 10.1038/s41467-019-13173-7 31727882 PMC6856176

[B70] Nakagawa Y. Johnson J. E. O’Leary D. D. (1999). Graded and areal expression patterns of regulatory genes and cadherins in embryonic neocortex independent of thalamocortical input. *J. Neurosci.* 19 10877–10885. 10.1523/JNEUROSCI.19-24-10877.1999 10594069 PMC6784968

[B71] Nau A. C. Pintar C. Arnoldussen A. Fisher C. (2015). Acquisition of Visual Perception in Blind Adults Using the BrainPort Artificial Vision Device. *Am. J. Occup. Ther.* 69 1–8. 10.5014/ajot.2015.011809 25553750 PMC4281706

[B72] Noordzij M. L. Zuidhoek S. Postma A. (2007). The influence of visual experience on visual and spatial imagery. *Perception* 36 101–112. 17357708 10.1068/p5390

[B73] Ockelford A. (2021). *Case studies in neuroplasticity: the musical abilities of some blind and autistic children.* London: KCL Neuroscience Society.

[B74] O’Leary D. D. Chou S.-J. Sahara S. (2007). Area patterning of the mammalian cortex. *Neuron* 56 252–269. 17964244 10.1016/j.neuron.2007.10.010

[B75] Page M. J. McKenzie J. E. Bossuyt P. M. Boutron I. Hoffmann T. C. Mulrow C. D. (2021). The PRISMA 2020 statement: an updated guideline for reporting systematic reviews. *BMJ* 372:n71. 10.1136/bmj.n71 PMC8005924 33782057

[B76] Pascual-Leone A. Hamilton R. (2001). The metamodal organization of the brain. *Progress Brain Res.* 134 427–445. 10.1016/s0079-6123(01)34028-1 11702559

[B77] Ptito M. Schneider F. C. G. Paulson O. B. Kupers R. (2008). Alterations of the visual pathways in congenital blindness. *Exp. Brain Res.* 187 41–49. 18224306 10.1007/s00221-008-1273-4

[B78] Pulvermuller F. (2018). Neural reuse of action perception circuits for language, concepts and communication. *Prog. Neurobiol.* 160 1–44. 10.1016/j.pneurobio.2017.07.001 28734837

[B79] Rakic P. (1988). Specification of cerebral cortical areas. *Science* 241 170–176. 10.1126/science.3291116 3291116

[B80] Rakic P. Suñer I. Williams R. W. (1991). A novel cytoarchitectonic area induced experimentally within the primate visual cortex. *Proc. Natl. Acad. Sci. U. S. A.* 88 2083–2087. 2006147 10.1073/pnas.88.6.2083 PMC51173

[B81] Raz N. Amedi A. Zohary E. (2005). V1 activation in congenitally blind humans is associated with episodic retrieval. *Cereb. Cortex* 15 1459–1468. 15647525 10.1093/cercor/bhi026

[B82] Reislev N. H. Dyrby T. B. Siebner H. R. Lundell H. Ptito M. Kupers R. (2017). Thalamocortical connectivity and microstructural changes in congenital and late blindness. *Neural Plast.* 2017:9807512. 10.1155/2017/9807512 PMC5366815 28386486

[B83] Renier L. Bruyer R. De Volder A. G. (2006). Vertical-horizontal illusion present for sighted but not early blind humans using auditory substitution of vision. *Percept. Psychophys.* 68 535–542. 16933419 10.3758/bf03208756

[B84] Renier L. De Volder A. G. (2010). Vision substitution and depth perception: Early blind subjects experience visual perspective through their ears. *Disabil. Rehabil.* 5 175–183. 10.3109/17483100903253936 20214472

[B85] Renier L. Laloyaux C. Collignon O. Tranduy D. Vanlierde A. Bruyer R. (2005). The Ponzo illusion with auditory substitution of vision in sighted and early-blind subjects. *Perception* 34 857–867. 16124271 10.1068/p5219

[B86] Révész G. Wolff H. A. (1950). *Psychology and Art of the Blind.* Harlow: Longmans.

[B87] Ring K. Cooper S. (1997). Near-death and out-of-body experiences in the blind: A study of apparent eyeless vision. *J. Near-Death Stud.* 16 101–147.

[B88] Ring K. Cooper S. (1999). *Mindsight: Near-death and out-of-body experiences in the blind. Mindsight: Near-death and out-of-body experiences in the blind.* Palo Alto, CA: William James for Center for Consciousness Studies.

[B89] Riso R. R. (1999). Strategies for providing upper extremity amputees with tactile and hand position feedback–moving closer to the bionic arm. *Technol. Health Care* 7 401–409. 10665673

[B90] Röder B. Rösler F. Hennighausen E. (1997). Different cortical activation patterns in blind and sighted humans during encoding and transformation of haptic images. *Psychophysiology* 34 292–307. 9175444 10.1111/j.1469-8986.1997.tb02400.x

[B91] Röder B. Rösler F. Neville H. J. (2000). Event-related potentials during auditory language processing in congenitally blind and sighted people. *Neuropsychologia* 38 1482–1502. 10906374 10.1016/s0028-3932(00)00057-9

[B92] Romains J. (1920). *La Vision extra-rétinienne et le sens paroptique, recherches de psycho-physiologie expérimentale et de physiologie histologique.* Paris: Nouvelle revue française.

[B93] Sadato N. Pascual-Leone A. Grafman J. Ibañez V. Deiber M. P. Dold G. (1996). Activation of the primary visual cortex by Braille reading in blind subjects. *Nature* 380 526–528. 8606771 10.1038/380526a0

[B94] Sampaio E. Maris S. Bach-y-Rita P. (2001). Brain plasticity: ‘visual’ acuity of blind persons via the tongue. *Brain Res.* 908 204–207. 11454331 10.1016/s0006-8993(01)02667-1

[B95] Siclari F. Valli K. Arnulf I. (2020). Dreams and nightmares in healthy adults and in patients with sleep and neurological disorders. *Lancet Neurol.* 19 849–859. 32949545 10.1016/S1474-4422(20)30275-1

[B96] Simor P. van der Wijk G. Nobili L. Peigneux P. (2020). The microstructure of REM sleep: Why phasic and tonic? *Sleep Med. Rev.* 52:101305. 10.1016/j.smrv.2020.101305 32259697

[B97] Starkiewicz W. Kuliszewski T. (1963). “The 80-channel elektroftalm,” in *Proceedings of the lnternational Congress Technology Blindness*, New York, NY.

[B98] Staunton H. O’Rourke K. (2012). The creation of a topographical world and its contents in the dreams of the congenitally blind. *Dreaming* 22 53–57.

[B99] Striem-Amit E. Amedi A. (2014). Visual cortex extrastriate body-selective area activation in congenitally blind people “Seeing” by using sounds. *Curr. Biol.* 24 687–692. 24613309 10.1016/j.cub.2014.02.010

[B100] Striem-Amit E. Dakwar O. Reich L. Amedi A. (2012). The large-scale organization of “visual” streams emerges without visual experience. *Cereb. Cortex* 22 1698–1709. 21940707 10.1093/cercor/bhr253

[B101] Striem-Amit E. Wang X. Bi Y. Caramazza A. (2018). Neural representation of visual concepts in people born blind. *Nat. Commun.* 9:5250. 10.1038/s41467-018-07574-3 PMC6286313 30531889

[B102] Timney B. Mitchell D. Giffin F. (1978). The development of vision in cats after extended periods of dark-rearing. *Exp. Brain Res.* 31 547–560. 658180 10.1007/BF00239811

[B103] Tinti C. Adenzato M. Tamietto M. Cornoldi C. (2006). Visual experience is not necessary for efficient survey spatial cognition: Evidence from blindness. *Q. J. Exp. Psychol.* 59 1306–1328. 10.1080/17470210500214275 16769626

[B104] Torrance E. P. Khatena J. Cunnington B. F. (1973). *Thinking Creatively with Sounds and Words: Sounds and Images, Onomatopoeia and Images: Directions Manual and Scoring Guide.* Lexington, MA: Personnel Press/Ginn.

[B105] Tyler M. Danilov Y. Bach-y-Rita P. (2003). Closing an open-loop control system: vestibular substitution through the tongue. *J. Integr. Neurosci.* 2 159–164. 15011268 10.1142/s0219635203000263

[B106] Vanlierde A. De Volder A. G. Wanet-Defalque M. C. Veraart C. (2003). Occipito-parietal cortex activation during visuo-spatial imagery in early blind humans. *Neuroimage* 19 698–709. 12880800 10.1016/s1053-8119(03)00153-8

[B107] Vanlierde A. Wanet-Defalque M. C. (2004). Abilities and strategies of blind and sighted subjects in visuo-spatial imagery. *Acta Psychol.* 116 205–222. 10.1016/j.actpsy.2004.03.001 15158183

[B108] Velázquez R. Bazán O. (2010). Preliminary evaluation of podotactile feedback in sighted and blind users. Conference proceedings: Annual International Conference of the IEEE Engineering in Medicine and Biology Society. *IEEE Eng. Med. Biol. Soc. Conf.* 2010 2103–2106. 10.1109/IEMBS.2010.5626205 21095950

[B109] Velázquez R. Bazán O. Magaña M. (2009). “A shoe-integrated tactile display for directional navigation,” in *Proceedings of the 2009 IEEE/RSJ International Conference on Intelligent Robots and Systems*, New York, NY.

[B110] Vetter P. Bola L. Reich L. Bennett M. Muckli L. Amedi A. (2020). Decoding natural sounds in early “visual” cortex of congenitally blind individuals. *Curr. Biol.* 30 3039–3044.e2. 32559449 10.1016/j.cub.2020.05.071 PMC7416107

[B111] Weeks R. Horwitz B. Aziz-Sultan A. Tian B. Wessinger C. M. Cohen L. G. (2000). A positron emission tomographic study of auditory localization in the congenitally blind. *J. Neurosci.* 20 2664–2672. 10729347 10.1523/JNEUROSCI.20-07-02664.2000 PMC6772250

