# Supplementary material for: Visuo-spatial imagery in dreams of congenitally and early blind: a systematic review
Source: Front Integr Neurosci. 2023 Jun 30;17:1204129. doi: 10.3389/fnint.2023.1204129 (PMC10347682; doi:10.3389/fnint.2023.1204129)
Supplement: Supplementary file 1 [file Table_1.docx]

**SUPPLEMENT**

**Table 1.** **Studies using neurotechnology to investigate the presence of visuo-spatial imagery in blind subjects during wake.**

| Authors and year | Participants: sample size, type of blindness | Main findings | Limitations |
| --- | --- | --- | --- |
| Röder et al. (1997) | 15 CB, 12 SC | EEG of blind participants during haptic perception and/or transformation of a haptic image  No differences in the performance between two groups.  A pronounced slow negative potential over the occipital cortex emerged only in CB subjects and was time-locked to the processing epochs. Its amplitude increased with the amount of processing load.  In both CB and SC: slow negative shift over 1) frontal cortex (beginning of each processing episode), 2) left central to parietal cortex (encoding and maintaining of haptic image) and 3) central to parietal cortex (image transformation). | Calculations to determine sample size are NR;  The EEG has only 18 electrodes in total, of which only 2 are from the occipital lobe (the main area investigated in this study).  7/15 CB subjects have rudimental light perception |
| Arno et al. (2001) | 6 EB, 6 SC | 6 EB and 6 SC were studied (PET) during three auditory processing tasks: a detection task with noise stimuli, a detection task with familiar sounds, and a pattern recognition task using the SSE. REST: higher activity in occipital cortex of EB compared to SC.  EFFECT OF TRAINING: left inferior frontal gyrus more activated during 2nd PET study (compared to 1st); Group x condition x day interaction not significant.  CONTRAST OF INTEREST (SSE - NOISE) masked with (SSE - REST): in SC, pattern recognition with SSE activated superior parietal lobule, inferior parietal lobule, and middle temporal gyrus (all right sided) during 1st PET study; right precuneus, right parietal lobule, and left inferior parietal lobule during 2nd PET study vs. in EB, it activated right and left precuneus and right middle temporal gyrus during 1st PET study; right middle frontal/precentral gyrus, left inferior parietal lobule, right and left precuneus, left precuneus, left middle occipital gyrus, left superior frontal gyrus, left inferior frontal gyrus, and superior parietal lobule during 2nd PET study; Group x condition interaction: left cuneus more activated in EB than in SC during 1st PET study (using SSE); right inferior occipital gyrus, right paracentralis lobule, and left inferior parietal lobule more activated in EB than in SC during 2nd PET study (using SSE) | Relatively small sample size  Male subjects only  Calculations to determine sample size are NR |
| De Volder et al. (2001) | 6 EB, 6 blindfolded SC | PET - regional cerebral blood flow was assessed in 6 EB and 6 SC during three conditions: resting state, passive listening to noise sounds, and mental imagery task (imagery of object shape) triggered by the sound of familiar objects.  Mental imagery vs. auditory processing control - 1) EB group: lateral occipito-temporal areas involved in mental imagery; no activation in prefrontal nor auditory cortex -  2) SC group: during mental imagery, activation of the same visual association areas as in the EB group -  3) Differences between EB and SC: activation in right prefrontal cortex and in left posterior cingular gyrus in SC group only; lower z scores in all activation foci (exception of the vermis) in the SC compared to the EB -  4) In both groups: no significant activation in the primary visual cortex nor in auditory regions. | Calculations to determine sample size are NR  Relatively small sample size  Male subjects only |
| Vanlierde et al. (2003) | 5 EB, 5 blindfolded SC | PET - regional cerebral blood flow was studied in 5 EB and 5 SC during visuo-spatial imagery who were instructed to generate a mental representation of verbally provided bidimensional patterns that were placed in a grid and to assess pattern symmetry in relation to a grid axis. This condition was contrasted with a verbal memory task  Differences between EB and SC subjects in visuo-spatial imagery activation contrasted to memory processing: No significantly higher activation in either group compared to the other;  Only very little trend for higher activation in right superior occipital gyrus in the EB subjects.  Conclusion: similar activation pattern in both groups during visuo-spatial imagery (also assessed by conjunction analysis); This activation pattern extended from precuneus towards superior parietal lobule bilaterally;  These cerebral regions were similarly activated in the visuo-spatial imagery task when contrasted with the 3 control conditions in both groups (conjunction analysis). Cerebral activation in both groups invloving the precuneus (BA 7), superior parietal lobule (BA 7), and occipital gyrus (BA 19) was similar during the visuo-spatial imagery task | Calculations to determine sample size are NR  Relatively small sample size  Only male subjects |
| Collignon et al. (2007) | 7 EB, 7 blindfolded SC | Study used repetitive transcranial magnetic stimulation (rTMS) to examine the functional role of right dorsal extrastriate cortex in the successful use of the PSVA. PSVA task: decrease in performance in real (compared to sham) stimulation condition in EB group only; only EB subjects required significantly longer exploration times in real (compared to sham) stimulation condition – In the EB, transient disruption of right dorsal extrastriate OC produces significant decrease in performance during PSVA use.  Auditory control tasks: 1) intensity discrimination task: EB subjects make significantly fewer errors than SC subjects (no rTMS effects for either group in this condition);  2) Pitch discrimination task: no significant rTMS effects in either group: 3) Location task: only EB subjects made significantly more errors in real (compared to sham) rTMS condition. | Calculations to determine sample size are NR;  One of the EB subjects become totally blind at the age of 7, and another one has diffuse light perception |
| Amedi et al. (2007) | 1 CB, 1 LB, 10 SC | (fMRI) Lateral-occipital tactile-visual area is activated in sighted, CB and LB subjects who recognize objects by extracting shape information from visual-to-auditory sensory substitution devices;  This region, the lateral-occipital tactile-visual area is not activated when subjects recognise objects by their typical sounds or when they simply learn to associate specific soundscapes with specific objects | Relatively small sample size: only 1 CB subject and 1 LB subject;  Calculations to determine sample size are NR; |
| Amedi et al. (2010) | 8 CB, 8 SC | (fMRI) TOR is characterized by robust LOC/lateral-occipital tactile-visual area activation in both CB and SC;  Pattern of fMRI activation during tactile exploration in LOC/ lateral-occipital tactile-visual area is bilateral in both CB and SC regardless of the palpating hand;  The CB group showed additional preferential activation in posterior occipital areas during TOR, compared to SC; Most prominent occipital activation during TOR was observed in the dorsal and central retinotopic areas bilaterally | Calculations to determine sample size are NR;  SMC was performed using the right hand only |
| Renier et al. (2010) | 12 EB, 12 blindfolded SC  6 SC | (fMRI) processing of auditory versus tactile and spatial versus nonspatial information in occipital cortex of the blind. During both auditory and tactile conditions: middle and inferior occipital gyri in the occipital cortex (OC) bilaterally activated in the EB group and de-activated in the SC group (no modality-specific activation in OC in neither EB nor SC group).  Contrast {Identification - Detection}: activation in frontal cortex in both groups, but OC activation in EB group only. {Localization - Detection}: activation in parietal cortex in both groups, but OC activation in EB subjects only.  {Detection - Localization} and {Detection - Identification}: no activation in OC in neither EB nor SC. {Localization - Identification}: part of middle occipital gyrus (MOG) more activated in EB subjects only. {Identification - Localization}: no activation in OC in neither EB nor SC. Significant correlation between performance accuracy and percentage of signal change in right MOG: observed during auditory localization task in EB subjects only.  No modality-specific OC activation was observed, the right middle occipital gyrus (MOG) showed a preference for spatial over nonspatial processing of both auditory and tactile stimuli. The occipital cortex of early-blind subjects is largely multisensory, specific regions retain the functional role they assume in sighted individuals. | Calculations to determine sample size are NR;  Tactile stimulation was performed using right hand only, but not all the subjects are right-handed.  The additional visual control group (6 SC) is not matched with CB group. |
| Striem-Amit et al. (2012) | 12 CB, 9 SC | (fMRI) 11 CB and 9 SC performing location versus form identification tasks following training on a sensory substitution device used for artificial vision. In both CB and SC: differentiation between dorsal and ventral pathways (processing of location and shapes, respectively).  CB group only: ventral visual cortex preference for shapes conveyed by sounds. Post hoc TASK contrasts: stream segregation between shape preference (ventral stream) and location preference (dorsal stream), independent of GROUP effect. GROUP effect of long-term blindness: increased involvement of posterior occipital cortex for processing soundscapes in CB (relative to).  Posterior ventral cortex: greater preference for shape in CB group only. All independent analyses (including RFX analyses in CB), for groups and individual subjects: clear dissociation between visual streams (regardless of task difficulty). | Calculations to determine sample size are NR;  SC subjects are not age-matched with CB subjects;  According to the published papers, the total number of participants is 20 (11 CB), while, in Table 1, the total number of participants is 21 (12 CB), the exclusion reason was NR |
| Striem-Amit and Amedi (2014) | 13 CB, 7 SC | (sensory substitution device + fMRI) In the CB: 1) extrastriate cortex is activated by sensory substitution sounds of body-shapes  2) the extrastriate body area cluster is the peak body-shape activity, and  3) the body-shape activity in the extrastriate body area shows anatomical consistency and selectivity; The body-shape activity exists regardless of visual experience, body expertise, or sensory input | Calculations to determine sample size are NR;  Baseline is just resting-state; Intrinsic functional-connectivity analysis only performed in the CB group (not in the SC);  The training duration is relatively long ,the brains of proficient users may have already undergone significant plastic changes due to the extensive use of sensory substitution devices (Ptito et al. 2005) |
| Striem-Amit et al. (2018) | 12 CB, 14 SC | fMRI to investigate how CB process concepts whose referents are imperceptible to them because of their visual nature (“rainbow”, “red”). Activity for these concepts was compared to that of sensorially-perceptible referents (“rain”), classical abstract concepts (“justice”) and concrete concepts (“cup”), providing a gradient between fully concrete and fully abstract concepts in the blind. Imperceptibility: in the CB, imperceptible concepts (e.g. “moon”, “island”, or “red”) positively correlated with activity in left dorsal anterior temporal lobe; In the SC, these concepts are perceptible and not correlated with activity in left dorsal anterior temporal lobe  Non-objecthood: in both CB and SC, abstract concepts (e.g. “freedom”) positively correlated with activity in lateral anterior temporal lobe (in the CB, concrete but imperceptible concepts such as “moon” NOT correlated with activity in lateral anterior temporal lobe).  Concreteness and perceptibility: in both CB and SC, concrete concepts (e.g. “cup”) correlated with activity in medial anterior temporal lobe (in the CB, activation in medial anterior temporal lobe shows preference for concrete and perceptible concepts such as “rain” over concrete but imperceptible concepts such as “moon”). Functional connectivity: in both CB and SC, dorsal and lateral anterior temporal lobe belong to same functional network (parts of dorsolateral anterior temporal lobe and inferior frontal lobe: areas which may be involved in processing semantic, non-sensorially derived information); In both CB and SC, medial anterior temporal lobe better connected to multisensory object-related regions in ventral visual cortex, as well as in frontal and parietal lobes | Calculations to determine sample size are NR;  Brain activity while listening to different concepts was compared between concepts and to baseline, but not to a “listening only” (sounds or words with no meaning) task |
| Mattioni et al. (2020) | 17 EB, 17 SC, 16 SC | fMRI to characterize Ventral Occipito-Temporal COrtex (VOTC) responses to eight categories presented acoustically in sighted and early blind individuals, and visually in a separate sighted group.  VOTC encodes sound categories in EB and SC subjects using representational structure and connectivity similar to the one found in vision  Sound categories: encoded more reliably in EB (using representational format closer to the one found in vision) than in SC | Calculations to determine sample size are NR;  7/17 of the EB subjects have "diffuse light perception";  In fMRI tasks, neither auditory nor visual controls: baseline is just rest, not meaningless sounds or images (sounds/images which are not associated with a specific thing) |

**Abbreviations:**

**CB**: Congenitally Blind (those who are born blind); **EB**: Early Blind (those who lost their sight before 5-7 years of age); **EEG**: Electroencephalography; **EMG**: Electromyography; **EOG**: Electrooculography; **fMRI**: functional Magnetic Resonance Imaging; **LB**: Late Blind (those who lost their sight after 5-7 years of age); **NR**: Not Reported; **NREM**: Non-Rapid Eye Movement; **PET**: Positron Emission Tomography; **PSVA**: Prosthesis Substituting Vision with Audition; **rTMS**: repetitive Transcranial Magnetic Stimulation; **SC**: Sighted Control.

**Table 2.** **Psychological investigations of visuo-spatial imagery in blind subjects.**

| Authors and year | Participants: sample size, type of blindness | Main findings | Limitations |
| --- | --- | --- | --- |
| Johnson (1980) | 91 CB and EB, 91 SC | Sensory image score obtained from blind and sighted participants  Blind subjects not restricted in the formation of visual images:  Blind group produced 210 visual images, SC group produced only 171 | Calculations to determine sample size NR  Exact number of CB and of EB subjects NR;  NR if investigators were blind to participants’ visual condition (blind or SC) when collecting and analysing data |
| Kerr (1983) | 10 CB (Exp 1-3), 10 SC (Exp 1,2), 10 SC (Exp 3) | Experiment 1 (subjects memorized the locations of several figures on a board and then were asked to form an image of the board and mentally to "scan" from one figure to another) - blind participants could preserve metric spatial information, but the response time was significantly longer for CB.  Experiment 2 (subjects required to form images of a "target" object, such as a radio, alongside a "context" object that was either very large (a car) or very small (a paper clip)) - Error rate: 3% (CB) and 5% (SC). Response times: CB slower (but not significantly) than SC; both CB and SC slower when context item was “car” than when it was “paper clip”  Experiment 3 (Subjects heard descriptions and were instructed to form images of scenes in which a target object was described in one of three relationships to the rest of the scene: spatially separated, contiguous but visually hidden or concealed, or contiguous and clearly visible) – Elapsed time: significant effects only for visual status (CB slower than SC). Difficulty of imagination: significant effects both for image type and for interaction of image type with visual status. Recall scores: significant effect for image type, but not for visual status or for the interaction. Overall recall scores did not differ for blind and sighted subjects, but sighted subjects reported forming the images significantly faster than did the blind | Calculations to determine sample size NR  NR if SC subjects were blindfolded during the experiments;  NR if investigators were blind to participants’ condition (CB or SC) when collecting and analysing dana; |
| Hollins (1985) | 1 CB, 1 EB, 4 LB, 4 SC with the eyes closed | 1. test: subjects had to imagine a checkerboard of which only some squares were filled. 2 test: same as 1., but pattern was 3D. Subjects had to name a common object that pattern resembled  Scoring: All blind subjects, and all but 1 SC able to correctly name at least 1 object  Number of correct spontaneous identifications ranged from 1 to 7 (average 2.2) in blind group, and from 0 to 6 (average of 3.0) in SC; No significant difference between averages of 2 groups.  Subjects performed better on multiple-choice portion of the tests: Number of correct identifications ranged from 4 to 10 (average 7.0) in blind group, and from 8 to 10 (average 9.2) in SC; No significant difference between averages of 2 groups. When points earned by spontaneous identifications added to points earned on multiple-choice items: scores ranged from 5 to 17 (average 9.2) in blind group, and from 9 to 16 (average 12.2) in SC; No significant difference between averages of 2 groups. Pictorial imagery ratio: in EB group, ratios range from 0.79 to 0.67; Negative correlation between pictorial imagery ratio and proportion of life spent without vision; In SC group, 1 subject obtained ratio in expected range (0.56), while the other 3 had much lower ratios (0.36, 0.38, 0.40). | Calculations to determine sample size NR  Small sample size;  CB, EB and LB subjects all part of the same group; Only 1 CB and 1 EB subjects. One blind subject lost vision due brain surgery. All blind subjects had various degrees of sight.  No metric to determine the similarity of objects to one another (eg dog vs horse); Impossible for investigators to be blind to participants’ visual condition (blind or SC) when collecting data; Investigators not blind to participants’ visual condition (blind or SC) when analysing data |
| Cornoldi et al. (1991) | 20 CB, 20 blindfolded SC, 15 CB,  15 blindfolded SC, 12 SC with eyes open; 32 blindfolded,SC | Experiment 1 (The subjects were told to follow mentally a pathway moving sequentially through a series of adjacent cubes) - CB and SC not significantly different, but group variable interacted significantly with dimensions: difficulty increased with complexity and with greater number of dimensions; increase in number of dimensions caused higher difficulty for CB than for SC (3D matrices: CB performance significantly lower than that of SC)  Experiment 2 (Same as 1, but the subjects were asked to transform an object along such dimensions as sweetness and temperature) – Spatial and verbal tasks: errors increased with complexity; increase greater for verbal than for spatial tasks. Difference between groups, and interaction between groups and dimensions: not significant  Experiment 3 (Same as 2, but with new groups) - Spatial and verbal tasks: errors increased with complexity; increase greater for verbal than for spatial tasks (see above - Experiment 2). 3D spatial patterns only: CB performance significantly lower than SC performance only. When a three-dimensional pattern exceeded sighted capacity, the blind and sighted showed similar patterns of errors. | Calculations to determine sample size NR  1 CB subject has large-form perception  Not possible to have investigators blind to participants’ condition (CB or SC) when collecting data  NR if investigators were blind to participants’ condition when analysing data |
| Heller et al. (1996) | 10 EB, 10 LB, 10 blindfolded SC, 20 SC | In three experiments the production and interpretation of perspective drawings was examined. They attempted to produce raised-line drawings of a surface at a number of angles—0° (panel horizontal), —22.5°, —45°, —67.5°, and —90° (vertical).  The three groups performed alike, both groups of blind subjects performed better than blindfolded sighted controls in experiment  Evidence of foreshortening only in the drawings of SC and LB subjects (not in those of EB subjects). LB had highest mean correct response (7.5), followed by SC (6.1) and by EB (5.8);  ANOVA indicated no significant difference between the 3 groups. Kruskal-Wallis: mean correct response differed significantly at -90°, but not at any other angle; blindfolded SC had difficulty discriminating -90° and -67.5° drawings; errors of CB less systematic, and more variable. | Calculations to determine sample size NR  Not possible to have investigators blind to participants’ condition (EB, LB or SC) when collecting data;  NR if investigators were blind to participants’ condition when analysing dana |
| Vecchi (1998) | 20 CB, 20 blindfolded SC | Congenitally blind and sighted participants were asked to memorise the spatial positions of target objects (cubes) in two -and three -dimensional matrices, while simultaneously performing a sequence of spatially based imagery operations  Both groups performed better in the 2D matrices than in the 3D matrices, but only CB group performed significantly worse in 3D matrix active (compared to 2D matrices, active and passive, and to 3D matrix passive), while SC group did not perform significantly differently in 2D vs. 3D matrices.  Performance of CB group significantly worse than that of SC group in all 4 conditions (2D matrix, active; 2D matrix, passive; 3D matrix, active; 3D matrix, passive).  In both groups, performance dropped modestly but significantly in articulatory suppression trials. The articulatory suppression affected both groups to the same extent, showing that CB did not use articulatory strategies any more than SC did | Calculations to determine sample size NR  Not possible to have investigators blind to participants’ condition (CB or SC) when collecting dana  NR if investigators were blind to participants’ condition when analysing data |
| Aleman et al. (2001) | 15 CB, 14 blindfolded SC | Pictorial task - subjects had to mentally compare the shape of the outline of three named objects and to indicate the odd one-out. Spatial task-the participants were asked to memorise the position of a target cube in two- and three-dimensional matrices, based on a sequence of spatially based imagery operations  1) Pictorial imagery task - CB performed worse than SC; both CB and SC performed significantly worse when tapping.  2) Spatial imagery task - CB performed worse than SC; both CB and SC performed worse when tapping. CB did not have more difficulty with pictorial than with spatial task, compared with SC. | Calculations to determine sample size NR  Impossible for investigators to be blind to participants’ visual condition when collecting dana |
| Vanlierde and Wanet-Defalque (2004) | 10 EB, 9 LB, 27 blindfolded SC | Subjects were instructed to generate a mental representation of verbally presented 2D patterns that were placed in a grid and to indicate how many pattern elements were in corresponding positions in the two halves of the grid according to a specific grid axis (vertical or horizontal). Three groups used different strategies  All SC, 9/10 LB and 1/10 EB subjects used "visual strategy"; 9/10 EB used "coordinate strategy"; 1 LB subject used mixed strategy (visual + coordinate); No difference in performance between group using visual strategy and group using coordinate strategy  Compare to experimenters, SC considered more patterns meaningful; EB considered more patterns meaningless.  Meaningful vs. meaningless patterns (i), vertical vs. horizontal symmetry (ii), intrinsically symmetric vs. non-symmetric pathways (iii), and first vs. second presentation of patterns (v): no difference in performance between the 3 subject groups. Intrinsic symmetry of pattern in accordance with extrinsic symmetry of matrix vs. intrinsic and extrinsic symmetries not in accordance (iv): SC and EB did better with symmetries in accordance; LB similar performance in accordance vs. not-accordance conditions. | Calculations to determine sample size NR  NR if only SC or all subjects were blindfolded;  Not possible to have investigators blind to participants’ condition (EB, LB or SC) when collecting data;  NR if investigators were blind to participants’ condition when analysing data. |
| Tinti et al. (2006) | 20 CB, 20 blindfolded SC, 13 CB, 13 LB, 13 blindfolded SC | Performance of persons with congenital blindness and that of blindfolded sighted persons on four survey representation-based tasks was analysed (1 Pathway A and B completion; 2 Direction estimates; 3 Straight-line distance judgements; 4 Pathway drawing. In experiment 2, LB group was included  Experiment 1: CB subjects (median= 3.7) more accurate than SC (median = 2.55); Drawings of CB group significantly more accurate than those of SC group, but difference only significant in drawings of pathway A.  Experiment 2: Drawings of CB (median = 3.7) and LB (median = 4.2) more accurate than those SC (median = 2.6).  Drawings of Pathway A and B analysed together: LB group performed better than SC; Difference between CB and SC groups, as well as difference between CB and LB groups, nonsignificant. Pathway A: Only difference between LB and SC group clearly significant. Pathway B: Drawings of LB group significantly more accurate than drawings of both CB and SC groups. | Calculations to determine sample size NR  Impossible for investigators to be blind to participants’ visual condition when collecting data; |
| Noordzij et al. (2007) | 12 EB, 15 LB, 15 blindfolded SC | Visual-imagery task - comparing object forms on the basis of a (verbally presented) object name. Spatial-imagery task - comparing angular differences on the basis of the position of clock hands on two clock faces (verbally presented clock times)  Visual imagery task - LB subjects made more errors than SC, but no difference between EB and LB, nor between EB and SC. No significant effects for the auditory-imagery task was revealed  Spatial imagery task – EB group made significantly more errors than SC, but no difference between EB and LB, nor between EB and SC; | Calculations to determine sample size NR  Investigators not blind to condition (EB, LB, SC) of participants when collecting data;  NR if investigators were blind to participants’ visual condition (EB, LB or SC) when analysing data |
| Cattaneo et al. (2010) | 13 CB and 3 EB  26 blindfolded SC | Testing whether the perceptual salience of vertical symmetry depends on visual experience  Both blind and sighted subjects remembered more accurately configurations that were symmetrical compared to those that were not. Sighted subjects displayed a higher level of facilitation by vertical than horizontal symmetry, but no such difference was found in the blind. This suggests that the perceptual salience of the vertical dimension is visually based | Calculations to determine sample size NR  Impossible for investigators to be blind to participants’ visual condition when collecting data;  NR if investigators were blind to participants’ visual condition (blind or SC) when analysing data; |

**Abbreviations:**

**CB**: Congenitally Blind (those who are born blind); **EB**: Early Blind (those who lost their sight before 5-7 years of age); **EEG**: Electroencephalography; **EMG**: Electromyography; **EOG**: Electrooculography; **fMRI**: functional Magnetic Resonance Imaging; **LB**: Late Blind (those who lost their sight after 5-7 years of age); **NR**: Not Reported; **NREM**: Non-Rapid Eye Movement; **PET**: Positron Emission Tomography; **PSVA**: Prosthesis Substituting Vision with Audition; **rTMS**: repetitive Transcranial Magnetic Stimulation; **SC**: Sighted Control.

**Table 3: Psychological investigations of visuo-spatial imagery in blind subjects.**

| Authors and year | Participants: sample size, type of blindness | Main findings | Limitations |
| --- | --- | --- | --- |
| Johnson (1980) | 91 CB and EB, 91 SC | Sensory image score obtained from blind and sighted participants  Blind subjects not restricted in the formation of visual images:  Blind group produced 210 visual images, SC group produced only 171 | Calculations to determine sample size NR  Exact number of CB and of EB subjects NR;  NR if investigators were blind to participants’ visual condition (blind or SC) when collecting and analysing data |
| Kerr (1983) | 10 CB (Exp 1-3), 10 SC (Exp 1,2), 10 SC (Exp 3) | Experiment 1 (subjects memorized the locations of several figures on a board and then were asked to form an image of the board and mentally to "scan" from one figure to another) - blind participants could preserve metric spatial information, but the response time was significantly longer for CB.  Experiment 2 (subjects required to form images of a "target" object, such as a radio, alongside a "context" object that was either very large (a car) or very small (a paper clip)) - Error rate: 3% (CB) and 5% (SC). Response times: CB slower (but not significantly) than SC; both CB and SC slower when context item was “car” than when it was “paper clip”  Experiment 3 (Subjects heard descriptions and were instructed to form images of scenes in which a target object was described in one of three relationships to the rest of the scene: spatially separated, contiguous but visually hidden or concealed, or contiguous and clearly visible) – Elapsed time: significant effects only for visual status (CB slower than SC). Difficulty of imagination: significant effects both for image type and for interaction of image type with visual status. Recall scores: significant effect for image type, but not for visual status or for the interaction. Overall recall scores did not differ for blind and sighted subjects, but sighted subjects reported forming the images significantly faster than did the blind | Calculations to determine sample size NR  NR if SC subjects were blindfolded during the experiments;  NR if investigators were blind to participants’ condition (CB or SC) when collecting and analysing dana; |
| Hollins (1985) | 1 CB, 1 EB, 4 LB, 4 SC with the eyes closed | 1. test: subjects had to imagine a checkerboard of which only some squares were filled. 2 test: same as 1., but pattern was 3D. Subjects had to name a common object that pattern resembled  Scoring: All blind subjects, and all but 1 SC able to correctly name at least 1 object  Number of correct spontaneous identifications ranged from 1 to 7 (average 2.2) in blind group, and from 0 to 6 (average of 3.0) in SC; No significant difference between averages of 2 groups.  Subjects performed better on multiple-choice portion of the tests: Number of correct identifications ranged from 4 to 10 (average 7.0) in blind group, and from 8 to 10 (average 9.2) in SC; No significant difference between averages of 2 groups. When points earned by spontaneous identifications added to points earned on multiple-choice items: scores ranged from 5 to 17 (average 9.2) in blind group, and from 9 to 16 (average 12.2) in SC; No significant difference between averages of 2 groups. Pictorial imagery ratio: in EB group, ratios range from 0.79 to 0.67; Negative correlation between pictorial imagery ratio and proportion of life spent without vision; In SC group, 1 subject obtained ratio in expected range (0.56), while the other 3 had much lower ratios (0.36, 0.38, 0.40). | Calculations to determine sample size NR  Small sample size;  CB, EB and LB subjects all part of the same group; Only 1 CB and 1 EB subjects. One blind subject lost vision due brain surgery. All blind subjects had various degrees of sight.  No metric to determine the similarity of objects to one another (eg dog vs horse); Impossible for investigators to be blind to participants’ visual condition (blind or SC) when collecting data; Investigators not blind to participants’ visual condition (blind or SC) when analysing data |
| Cornoldi et al. (1991) | 20 CB, 20 blindfolded SC, 15 CB,  15 blindfolded SC, 12 SC with eyes open; 32 blindfolded,SC | Experiment 1 (The subjects were told to follow mentally a pathway moving sequentially through a series of adjacent cubes) - CB and SC not significantly different, but group variable interacted significantly with dimensions: difficulty increased with complexity and with greater number of dimensions; increase in number of dimensions caused higher difficulty for CB than for SC (3D matrices: CB performance significantly lower than that of SC)  Experiment 2 (Same as 1, but the subjects were asked to transform an object along such dimensions as sweetness and temperature) – Spatial and verbal tasks: errors increased with complexity; increase greater for verbal than for spatial tasks. Difference between groups, and interaction between groups and dimensions: not significant  Experiment 3 (Same as 2, but with new groups) - Spatial and verbal tasks: errors increased with complexity; increase greater for verbal than for spatial tasks (see above - Experiment 2). 3D spatial patterns only: CB performance significantly lower than SC performance only. When a three-dimensional pattern exceeded sighted capacity, the blind and sighted showed similar patterns of errors. | Calculations to determine sample size NR  1 CB subject has large-form perception  Not possible to have investigators blind to participants’ condition (CB or SC) when collecting data  NR if investigators were blind to participants’ condition when analysing data |
| Heller et al. (1996) | 10 EB, 10 LB, 10 blindfolded SC, 20 SC | In three experiments the production and interpretation of perspective drawings was examined. They attempted to produce raised-line drawings of a surface at a number of angles—0° (panel horizontal), —22.5°, —45°, —67.5°, and —90° (vertical).  The three groups performed alike, both groups of blind subjects performed better than blindfolded sighted controls in experiment  Evidence of foreshortening only in the drawings of SC and LB subjects (not in those of EB subjects). LB had highest mean correct response (7.5), followed by SC (6.1) and by EB (5.8);  ANOVA indicated no significant difference between the 3 groups. Kruskal-Wallis: mean correct response differed significantly at -90°, but not at any other angle; blindfolded SC had difficulty discriminating -90° and -67.5° drawings; errors of CB less systematic, and more variable. | Calculations to determine sample size NR  Not possible to have investigators blind to participants’ condition (EB, LB or SC) when collecting data;  NR if investigators were blind to participants’ condition when analysing dana |
| Vecchi (1998) | 20 CB, 20 blindfolded SC | Congenitally blind and sighted participants were asked to memorise the spatial positions of target objects (cubes) in two -and three -dimensional matrices, while simultaneously performing a sequence of spatially based imagery operations  Both groups performed better in the 2D matrices than in the 3D matrices, but only CB group performed significantly worse in 3D matrix active (compared to 2D matrices, active and passive, and to 3D matrix passive), while SC group did not perform significantly differently in 2D vs. 3D matrices.  Performance of CB group significantly worse than that of SC group in all 4 conditions (2D matrix, active; 2D matrix, passive; 3D matrix, active; 3D matrix, passive).  In both groups, performance dropped modestly but significantly in articulatory suppression trials. The articulatory suppression affected both groups to the same extent, showing that CB did not use articulatory strategies any more than SC did | Calculations to determine sample size NR  Not possible to have investigators blind to participants’ condition (CB or SC) when collecting dana  NR if investigators were blind to participants’ condition when analysing data |
| Aleman et al. (2001) | 15 CB, 14 blindfolded SC | Pictorial task - subjects had to mentally compare the shape of the outline of three named objects and to indicate the odd one-out. Spatial task-the participants were asked to memorise the position of a target cube in two- and three-dimensional matrices, based on a sequence of spatially based imagery operations  1) Pictorial imagery task - CB performed worse than SC; both CB and SC performed significantly worse when tapping.  2) Spatial imagery task - CB performed worse than SC; both CB and SC performed worse when tapping. CB did not have more difficulty with pictorial than with spatial task, compared with SC. | Calculations to determine sample size NR  Impossible for investigators to be blind to participants’ visual condition when collecting dana |
| Vanlierde and Wanet-Defalque (2004) | 10 EB, 9 LB, 27 blindfolded SC | Subjects were instructed to generate a mental representation of verbally presented 2D patterns that were placed in a grid and to indicate how many pattern elements were in corresponding positions in the two halves of the grid according to a specific grid axis (vertical or horizontal). Three groups used different strategies  All SC, 9/10 LB and 1/10 EB subjects used "visual strategy"; 9/10 EB used "coordinate strategy"; 1 LB subject used mixed strategy (visual + coordinate); No difference in performance between group using visual strategy and group using coordinate strategy  Compare to experimenters, SC considered more patterns meaningful; EB considered more patterns meaningless.  Meaningful vs. meaningless patterns (i), vertical vs. horizontal symmetry (ii), intrinsically symmetric vs. non-symmetric pathways (iii), and first vs. second presentation of patterns (v): no difference in performance between the 3 subject groups. Intrinsic symmetry of pattern in accordance with extrinsic symmetry of matrix vs. intrinsic and extrinsic symmetries not in accordance (iv): SC and EB did better with symmetries in accordance; LB similar performance in accordance vs. not-accordance conditions. | Calculations to determine sample size NR  NR if only SC or all subjects were blindfolded;  Not possible to have investigators blind to participants’ condition (EB, LB or SC) when collecting data;  NR if investigators were blind to participants’ condition when analysing data. |
| Tinti et al. (2006) | 20 CB, 20 blindfolded SC, 13 CB, 13 LB, 13 blindfolded SC | Performance of persons with congenital blindness and that of blindfolded sighted persons on four survey representation-based tasks was analysed (1 Pathway A and B completion; 2 Direction estimates; 3 Straight-line distance judgements; 4 Pathway drawing. In experiment 2, LB group was included  Experiment 1: CB subjects (median= 3.7) more accurate than SC (median = 2.55); Drawings of CB group significantly more accurate than those of SC group, but difference only significant in drawings of pathway A.  Experiment 2: Drawings of CB (median = 3.7) and LB (median = 4.2) more accurate than those SC (median = 2.6).  Drawings of Pathway A and B analysed together: LB group performed better than SC; Difference between CB and SC groups, as well as difference between CB and LB groups, nonsignificant. Pathway A: Only difference between LB and SC group clearly significant. Pathway B: Drawings of LB group significantly more accurate than drawings of both CB and SC groups. | Calculations to determine sample size NR  Impossible for investigators to be blind to participants’ visual condition when collecting data; |
| Noordzij et al. (2007) | 12 EB, 15 LB, 15 blindfolded SC | Visual-imagery task - comparing object forms on the basis of a (verbally presented) object name. Spatial-imagery task - comparing angular differences on the basis of the position of clock hands on two clock faces (verbally presented clock times)  Visual imagery task - LB subjects made more errors than SC, but no difference between EB and LB, nor between EB and SC. No significant effects for the auditory-imagery task was revealed  Spatial imagery task – EB group made significantly more errors than SC, but no difference between EB and LB, nor between EB and SC; | Calculations to determine sample size NR  Investigators not blind to condition (EB, LB, SC) of participants when collecting data;  NR if investigators were blind to participants’ visual condition (EB, LB or SC) when analysing data |
| Cattaneo et al. (2010) | 13 CB and 3 EB  26 blindfolded SC | Testing whether the perceptual salience of vertical symmetry depends on visual experience  Both blind and sighted subjects remembered more accurately configurations that were symmetrical compared to those that were not. Sighted subjects displayed a higher level of facilitation by vertical than horizontal symmetry, but no such difference was found in the blind. This suggests that the perceptual salience of the vertical dimension is visually based | Calculations to determine sample size NR  Impossible for investigators to be blind to participants’ visual condition when collecting data;  NR if investigators were blind to participants’ visual condition (blind or SC) when analysing data; |

**Abbreviations:**

**CB**: Congenitally Blind (those who are born blind); **EB**: Early Blind (those who lost their sight before 5-7 years of age); **EEG**: Electroencephalography; **EMG**: Electromyography; **EOG**: Electrooculography; **fMRI**: functional Magnetic Resonance Imaging; **LB**: Late Blind (those who lost their sight after 5-7 years of age); **NR**: Not Reported; **NREM**: Non-Rapid Eye Movement; **PET**: Positron Emission Tomography; **PSVA**: Prosthesis Substituting Vision with Audition; **rTMS**: repetitive Transcranial Magnetic Stimulation; **SC**: Sighted Control.

**Table 4. Studies investigating visuo-spatial imagery during near-death and out-of-body experiences in blind subjects.**

| Authors and year | Participants: sample size, type of blindness | Main findings | Limitations |
| --- | --- | --- | --- |
| Ring and Cooper (1997) | 14 EB, 11 LB, 6 visually impaired subjects | 25 subjects (80%) claimed sight during either an NDE or an OBE: 9/14 (64%) of the EB subjects claimed sight during either an NDE or an OBE.  Of 21 EB, LB or visually impaired subjects reporting an NDE, 15 claimed to have some visual experience (5/10 EB subject who had an NDE reported vision): 10/21 saw their physical body, 8/21 went through a tunnel or dark space, 8/21 saw a radiant light - No difference in the frequency of these elements between the 3 subject groups.  9/10 subjects who reported an OBE claimed sight (7/10 reported seeing their body). When comparing NDEs/OBEs with their dreams, EB subjects report visual imagery in the NDEs/OBEs only. | Calculations to determine sample size NR  Investigators were not blind to participants’ condition (EB, LB or visually impaired) when collecting and analysing data.  Possibly more than 9/14 EB subjects had visual experiences during their NDEs/OBEs, but did not know how to describe them. As suggested by the authors themselves, subjects may be using visual terms to describe experiences that are not visual, but rather supramodal (“transcendental awareness”) |

**Abbreviations:**

**CB**: Congenitally Blind (those who are born blind); **EB**: Early Blind (those who lost their sight before 5-7 years of age); **NDE:** Near death experience; **OBE**: out-of-body experiences; **SC**: Sighted Control.

**Table 5. Characteristics and main findings of studies investigating the presence of visuo-spatial imagery in blind subjects’ dreams.**

| Authors and year | Participants: sample size, type of blindness | Main findings | Limitations |
| --- | --- | --- | --- |
| Berger et al. (1962) | 3 CB, 6 LB | No REMs during sleep and no visual imagery in the dreams of the CB and of the LB who have been blind for 30 or more years;  REMs during sleep and visual imagery in the dreams of the LB who have been blind for 15 years or less | Small sample size, no female participants and no control group  EEG only for two nights  Techniques and measures employed to collect and analyse dream reports are NR  Fisher (1965)*: the failure to detect REMs in the blind could be due to the EOG failing to detect eye movements in subjects with retrolental fibroplasia (extensive destruction of the retina) |
| Amadeo and Gomez (1966) | 3 CB | All subjects had cyclically recurring patterns of EEG and EMG activity during sleep  All subjects showed clearly defined rapid eye movements during REM sleep, even if these rapid eye movements were fewer and of lesser amplitude than those observed in sighted subjects in other studies;  Subjects only reported dreams upon awakenings during sleep stage 2 (1/5 times) and during REM sleep (7/7 times)  No visual imagery in the subjects' dreams, but only auditory, tactile, proprioceptive and, to a lesser extent, olfactory imagery | Small sample size and no control group  EEG, EOG and EMG only for two nights; EEG only had 8 channels  Dream reports upon awakening only for 1 night;  The "detailed inquiry" employed by the experimenter to assess the presence of sensory imagery in the participants' dreams is NR |
| Kerr et al. (1982) | 4 CB, 2 LB, 4 SC | No rapid eye movements in the blind, but phasic REM sleep detectable (sawtooth waves)  In both SCs and blind participants, more dreams reported during REM sleep than during NREM sleep or at sleep onset  Overall, blind and sighted subjects have similar dream content  In the dreams of the CB who are totally blind: no visual imagery, but spatial representations  In the dreams of the CB with residual form vision: some visual imagery (comparable to what they can see during wakefulness);  In the dreams of the LB: visual imagery, even in dreams about people/object only encountered after blindness onset | Calculations to determine sample size NR; Small sample size  Only one CB participant with no residual vision of any kind; Only one male participant  Impossible for investigators to be blind to participants’ visual condition (blind or SC) when collecting data;  Investigators not blind to participants’ visual condition (blind or SC) when analysing dana  Fisher (1965)*: the failure to detect REMs in the blind could be due to the EOG failing to detect eye movements in subjects with retrolental fibroplasia (extensive destruction of the retina) |
| Hurovitz et al. (1999) | 7 CB, 8LB, SC: Hall and Van de Castle (1966)* norms | Positive correlation between lack of visual oneiric imagery and 1) congenital or early blindness, 2) total blindness, and 3) a high percentage of the person's total life spent being blind;  One of the early blind participants (totally blind since the age of 4) reported visual imagery (not metaphorical) in his dreams;  Compared to Hall and Van de Castle norms*, significantly more frequent auditory and haptic/gustatory/olfactory impressions in the dreams of participants (both CB and LB) with little or no oneiric visual imagery, but significantly more frequent visual imagery and significantly less frequent auditory and haptic/gustatory/olfactory impressions in the dreams of two LB participants | Calculations to determine sample size NR; They do not have their own control group;  Cause of blindness of the participants are NR;  Investigators not blind to participants’ visual condition (CB, LB or SC) when collecting and analysing data |
|  |  |  |  |
|  |  |  |  |
| Holzinger (2000) | 12 CB, 11 LB blind before 14 years, 11 LB blind after 14 years, 21 SC | No visual imagery in the dreams of the CB; Some visual imagery (including colours in 6 dreams) in the dreams of those who lost sight before 14 years of age;  Frequent visual imagery (including colours) in the dreams of those who lost sight after 14 years of age;  Visual imagery always present in the dreams of the SC  More auditory and tactile imagery in the dreams of the CB than in those of the SC;  Gustatory and olfactory imagery rare in the dreams of all 4 groups | Calculations to determine sample size NR  Cause of blindness of the participants are NR  NR if investigators were blind to participants’ visual condition (CB, LB or SC) when collecting and analysing data;  Methods employed to collect and analyse dream reports are NR |
| Bértolo et al., (2003) | 10 CB, 9 SC | Visual imagery in CB subjects, both in dreams and when awake;  No differences between CB and SC in the graphical representation of dreams;  CB subjects had a lower rate of dream recall than SC;  Both CB and SC had significant negative correlations between the Visual Activity Index and alpha power in the central and occipital O2 derivations | Calculations to determine sample size NR  Polysomnography and dream reports only for two nights (8 awakenings/subject in total); EEG only had 8 channels;  Impossible for investigators to be blind to participants’ visual condition (CB or SC) when collecting data;  When calculating the Visual Activity Index, the authors did not consider that visual terminology can sometimes be used metaphorically (Kerr & Domhoff, 2004);  The authors assumed that alpha attenuation in CB subjects is correlated with visual imagery: not considering potential cross-modal plasticity of the “blind” occipital cortex (Cohen et al., 1997; Kujala et al., 2005; Kupers et al., 2006); The authors did not consider that an "overall decrease in alpha power" has been reported over the occipital cortex of blind subjects during wakefulness (Aubin et al., 2018) |
| Staunton and O'Rourke (2012) | 8 CB, 1 CB female college student whose 15 dreams were retrieved from DreamBank*, SC: 1000 dreams collected from American college students by Hall and Van de Castle (1966)** | Most frequently experienced perceptual modality in the dreams of the CB is auditory, followed (in decreasing order) by somaesthetic, olfactory, gustatory, pain and temperature  No visual imagery in the dreams of the CB  No significant differences in the 4/10 Hall and Van de Castle categories (setting, objects, characters and activities) between CB and SC | Calculations to determine sample size NR;  Small dream sample size (n = 22);  Only one male participant  They do not have their own control group;  NR if investigators were blind to participants’ visual condition (CB or SC) when analysing data;  Presence of visual imagery not directly assessed (just assumed to be absent in the dreams of the CB) |
|  |  |  |  |
| Meaidi et al. (2014) | 11 CB, 14 LB, 25 SC | Blind participants (CB and LB) had fewer visual dream impressions than SC participants;  No visual impressions in the dreams of the CB without residual vision, but some visual impressions (colour, light) in the dreams of the 3 CB subjects with residual vision  In LB participants: average blindness duration index negatively correlated with duration, clarity, and color content of visual dream impressions  More auditory, tactile, gustatory, and olfactory dream components in the CB group (compared to SC)  More tactile dream impressions in the LB group (compared to SC) | Calculations to determine sample size NR  3 of the CB participants have residual vision (potential confounder);  Vividness of Visual Imagery Questionnaire only in LB and SC participants;  NR if investigators were blind to participants’ visual condition (CB, LB or SC) when analysing data; |
|  |  |  |  |
| Bértolo et al. (2017) | 10 CB, 10 SC | CB and SC subjects have the same dream recall ability upon awakening (60%);  Higher REM density in the SC group; Less frequent REMs in the CB group;  No differences regarding REM density and recall ability;  No differences in neither the Global Activity Index nor the Visual Activity Index between the CB and the SC groups: visual, auditory, tactile and kinaesthetic imagery in the dreams of both groups. | Calculations to determine sample size NR; Dream reports and Polysomnography only for two nights  Cause of blindness of the participants are NR  Impossible for investigators to be blind to participants’ visual condition (CB or SC) when collecting data;  NR if investigators were blind to participants’ visual condition (CB or SC) when analysing data;  When calculating the Visual Activity Index, the authors did not consider that visual terminology can sometimes be used metaphorically (Kerr & Domhoff, 2004). |
| Christensen et al. (2019) | 5 CB, 6 LB, 11 SC | Both the CB and LB groups had a significantly lower eye movements coverage than the SC group;  CB participants reported experiencing a visual element in one of their dreams on two occasions, but in general the CB group had very few or no visual dream elements, compared to the LB and SC groups;  Negative correlation between Blind Duration Index and visual dream content;  Positive correlation between frequency of visual dream content and eye movement coverage in both REM and NREM sleep | Calculations to determine sample size NR;  Polysomnography only for two nights;  Impossible for investigators to be blind to participants’ visual condition (CB, LB or SC) when collecting dana  NR if investigators were blind to participants’ visual condition (CB, LB or SC) when analysing data  Fisher (1965)***: the failure to detect REMs in the blind could be due to the EOG failing to detect eye movements in subjects with retrolental fibroplasia (extensive destruction of the retina) |

**Abbreviations:**

**CB**: Congenitally Blind (those who are born blind); **EB**: Early Blind (those who lost their sight before 5-7 years of age); **EEG**: Electroencephalography; **EMG**: Electromyography; **EOG**: Electrooculography; **LB**: Late Blind (those who lost their sight after 5-7 years of age); **NR**: Not Reported; **NREM**: Non-Rapid Eye Movement; **REM**: Rapid Eye Movement; **SC**: Sighted Control.
